# Supplementary material for: Germ cell differentiation requires Tdrd7-dependent chromatin and transcriptome reprogramming marked by germ plasm relocalization
Source: Dev Cell. 2021 Mar 8;56(5):641–656.e5. doi: 10.1016/j.devcel.2021.02.007 (PMC7957325; doi:10.1016/j.devcel.2021.02.007)
Supplement: Document S1. Figures S1–S6 [file mmc1.pdf]

Developmental Cell, Volume *56*

Supplemental information

Germ cell differentiation requires Tdrd7-dependent chromatin and transcriptome reprogramming marked by germ plasm relocalization

Fabio M. D'Orazio, Piotr J. Balwierz, Ada Jimenez González, Yixuan Guo, Benjamín Hernández-Rodríguez, Lucy Wheatley, Aleksandra Jasiulewicz, Yavor Hadzhiev, Juan M. Vaquerizas, Bradley Cairns, Boris Lenhard, and Ferenc Müller

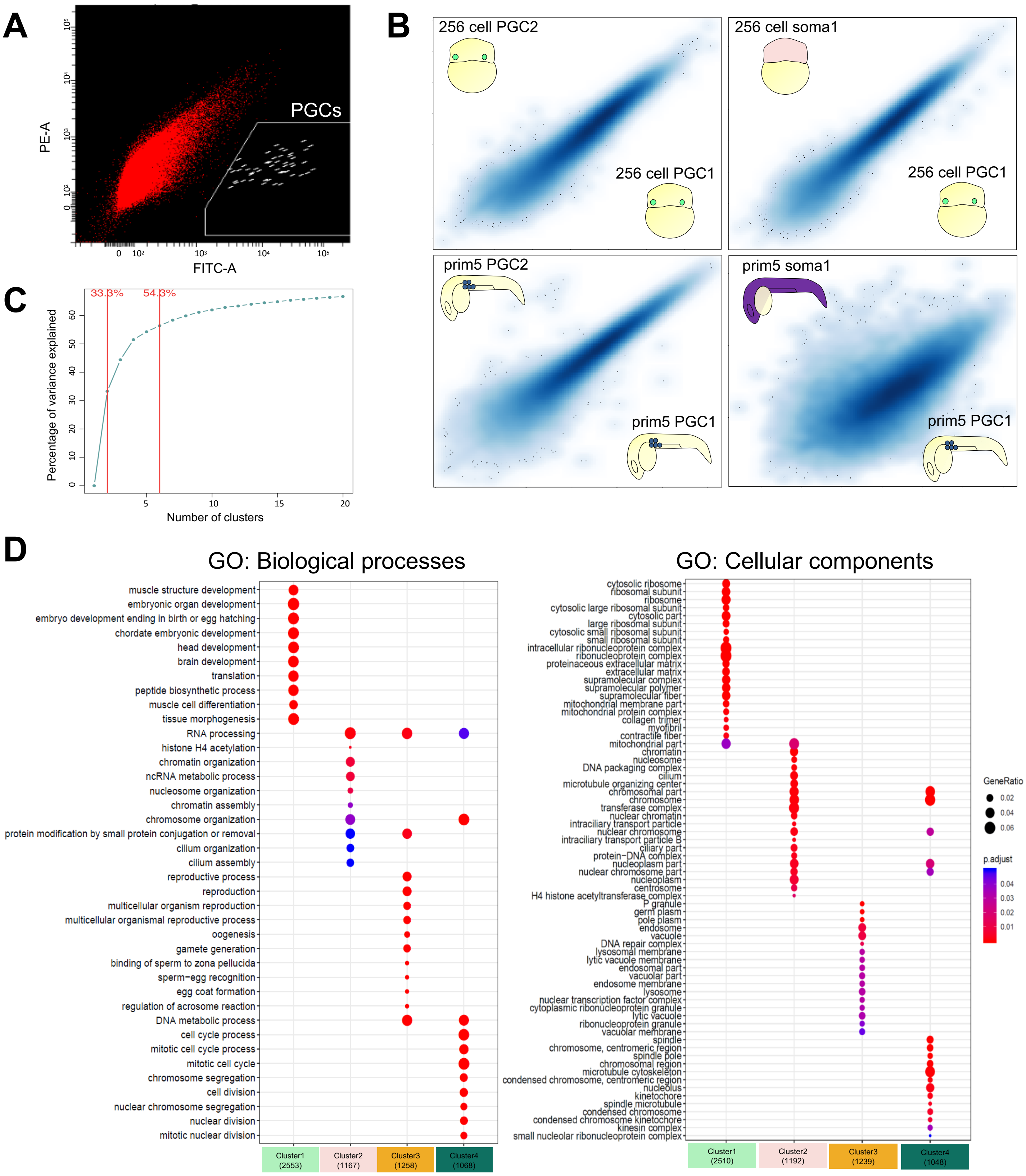

Supplementary Figure 1. **Characterisation of PGC transcriptome highlights early developmental similarities and late divergence between PGCs and somatic cells. Related to Figure 1** (A) FACS profile of GFP-expressing PGCs at prim-5 stage. GFP-positive and –negative cells are shown in white and red respectively. (B) Transcriptome correlation profiles after RNA-seq reads normalisation. Read counts for each gene were normalised for library size and gene length (tpm) and correlation among stages and cell types was compared. Reads are shown as Log_2(tpm+1). (C) Justification of number of k-means selected based on inter-sample variance. (D) Biological processes and cellular components GO analysis for the four identified expression groups by k-mean clustering (p-adjusted < 0.05).

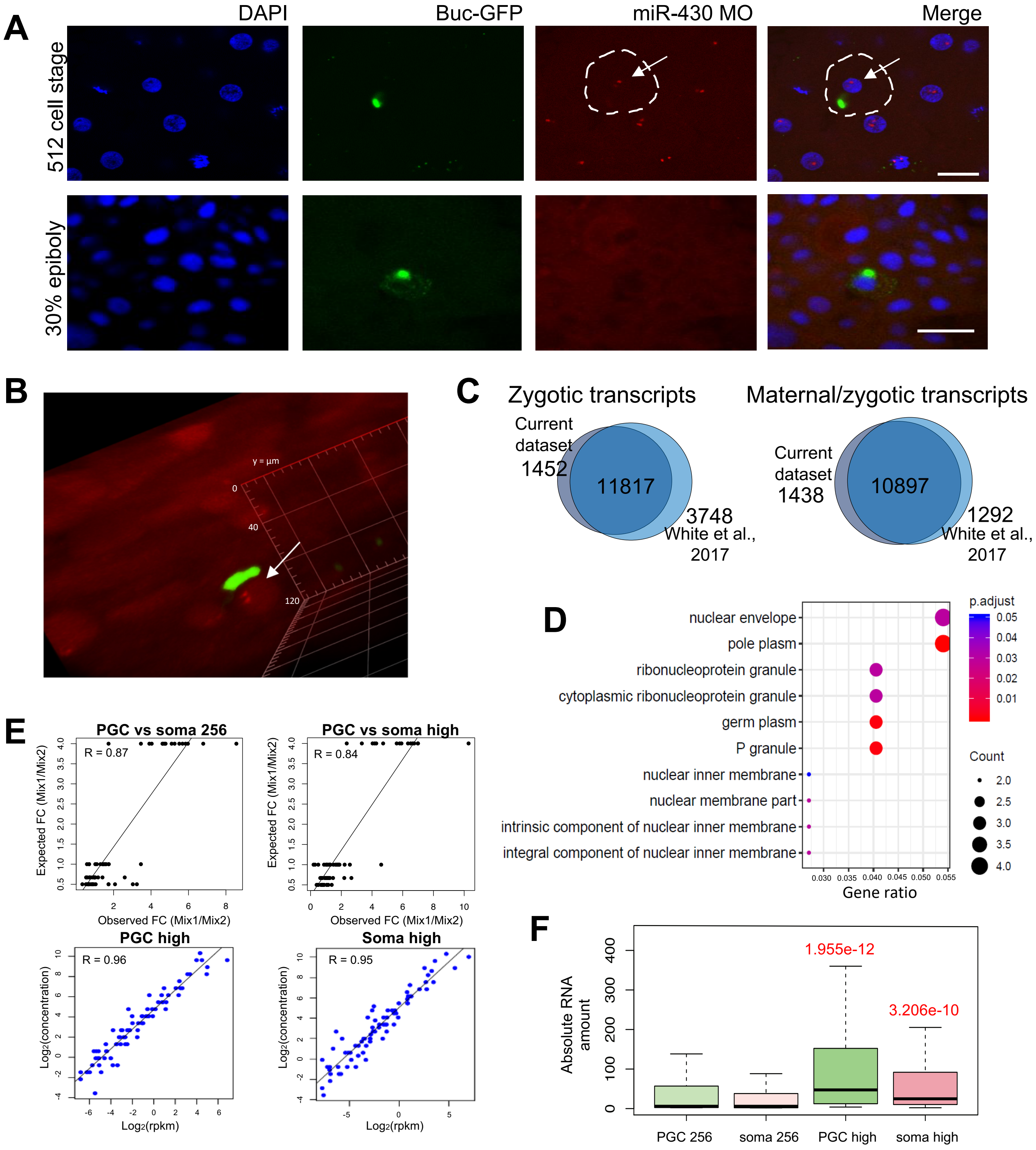

Supplementary Figure 2. PGCs do not delay transcriptional activation. Related to Figure 2 (A) Maximum intensity projection of a multi-stack image of *miR-430* transcriptional activity at 512-cell stage and 30% epiboly. PGC boundaries are highlighted as white, dashed lines. Arrow indicates the nucleus of a germ plasm-carrying cell where transcriptional foci are detected. Scale bar is 30 µm. Number of embryos, n = 19 and 31 respectively. (B) 3D rendering of a *miR-430*-expressing nucleus (arrow) and a GFP-tagged germ granule. White arrow indicates the germ cell nucleus. Number of embryos, n = 24. (C) Venn diagram for predicted zygotic/maternal and zygotic genes from two independent RNA-seq datasets. (D) Cellular components GO analysis for genes upregulated in PGCs after the first wave of ZGA (from 256-cell to high stage). P-adjusted < 0.05. (E) Representative regression scatter plots between expected vs observed ERCC mix ratios (top) and ERCC concentrations vs ERCC reads (bottom). Tpm threshold for normalised ERCC reads is 1. (F) Absolute RNA concentration normalised to internal control (ERCC spike-in) for genes upregulated in germ plasm-carrying cells between 256-cell and high stage. Numbers show p-values calculated by Wilcoxon test.

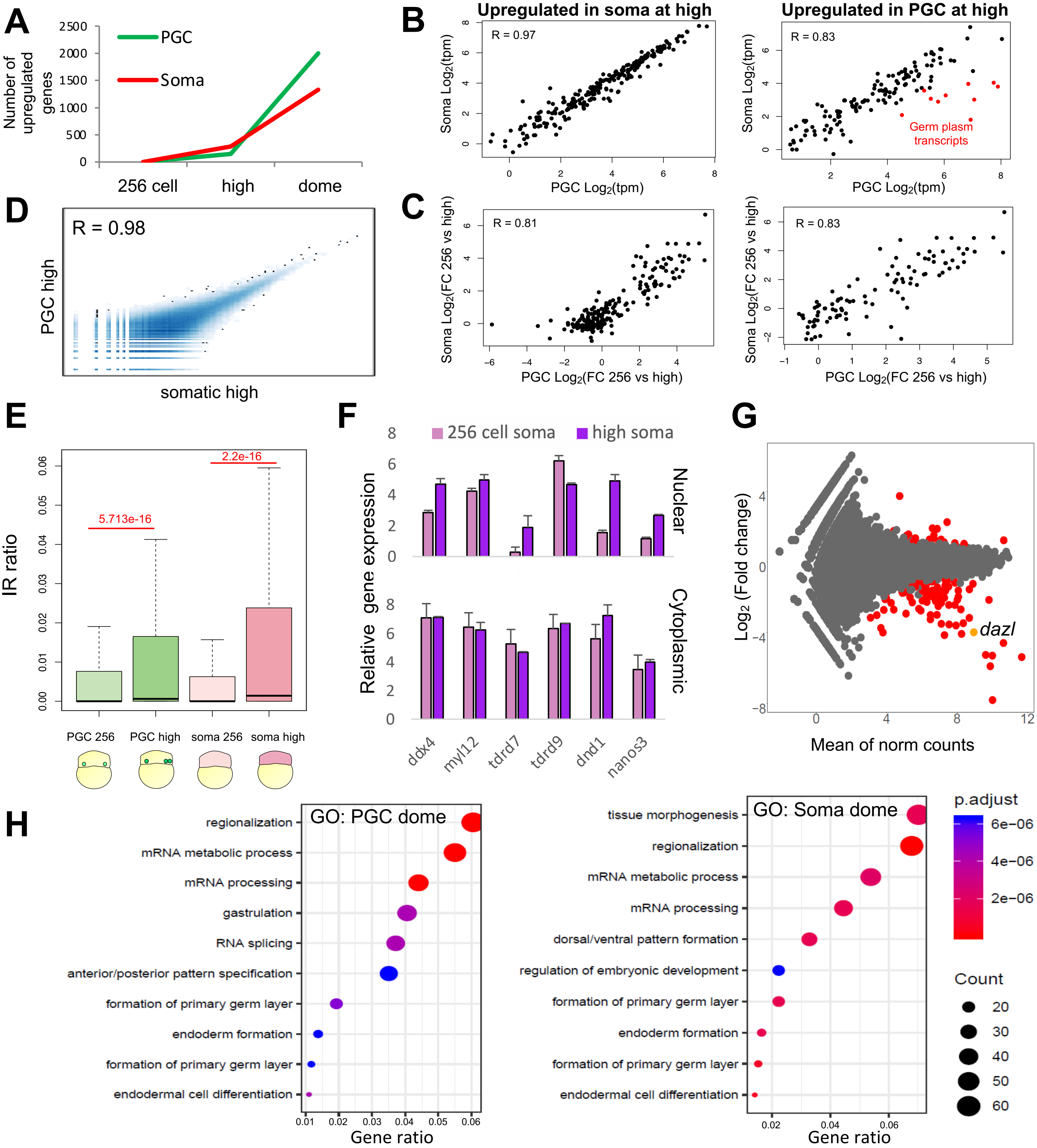

Supplementary Figure 3. Differential transcriptome between PGCs and somatic cells at early stages is not caused by differential transcription. Related to Figure 3 (A) Count of upregulated genes from previous stage in PGCs and somatic cells at high and dome stages. (B) Correlation of normalised RNA-seq reads (tpm) for genes upregulated from the previous stage in the PGCs or somatic cells only at high stage. Germ plasm genes are in red. (C) Correlation between fold change increases from 256-cell to high stages in PGCs and somatic cells for gene subsets. (D) Correlation plot for open chromatin regions detected in PGCs and somatic cells at high stage. (E) Intron retention ratio for all transcripts in PGCs (green) and somatic cells (purple) before and after ZGA. T-test was used to calculate p-values (in red). (F) Relative gene expression fold change normalised to a reference gene in somatic cells for nuclear and cytoplasmic fractions (number of replicates = 2). Error bars show standard errors. (G) Differential expressed genes between 256-cell and high stages in PGCs. Significantly differentially expressed genes are in red. *dazl* is in orange. P adjusted < 0.1, $\log_2$FC < -1/> 1. (H) Biological processes GO analysis for genes upregulated from high to dome.

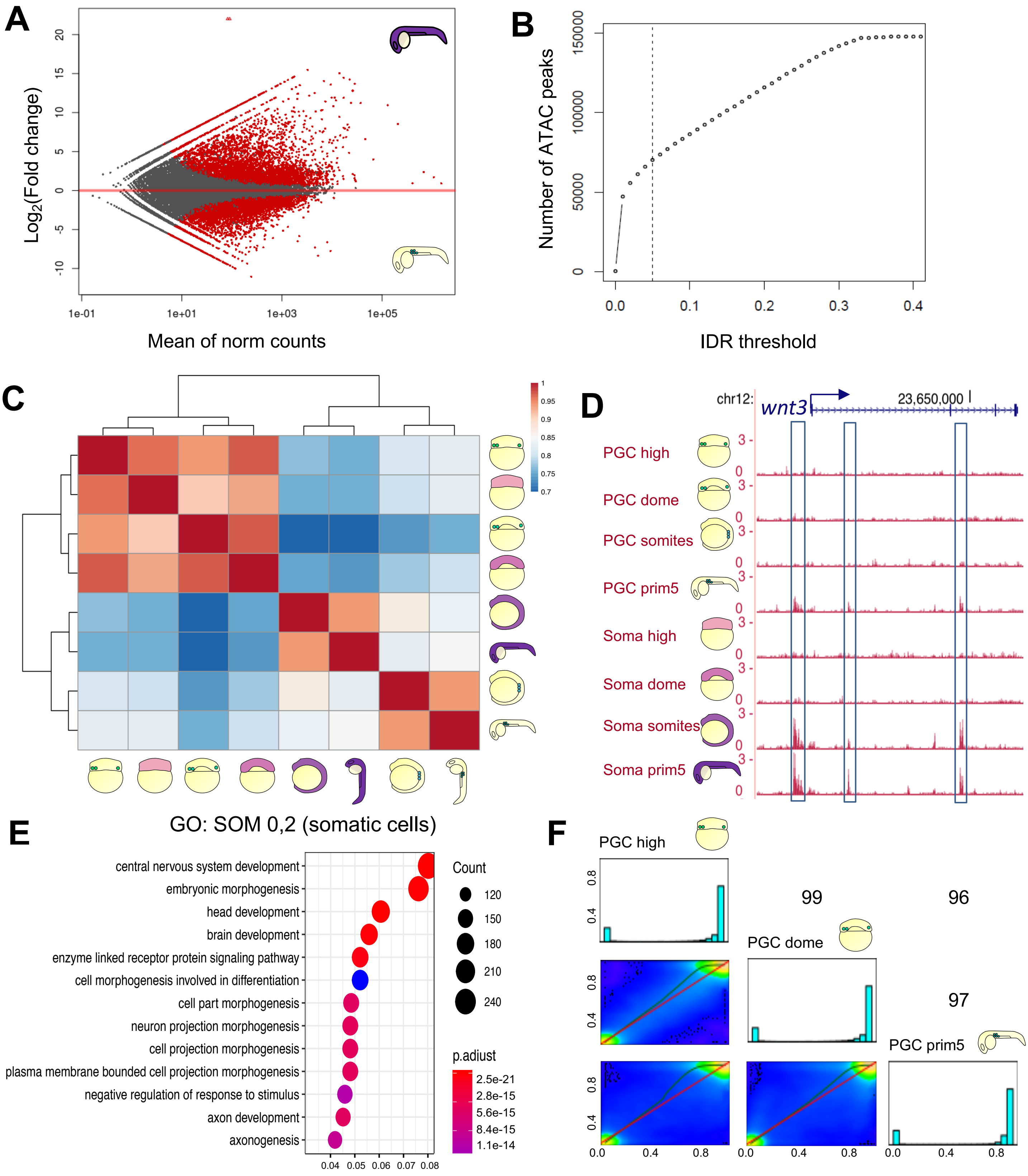

Supplementary Figure 4. Gradual acquisition of germ identity is accompanied by epigenetic changes. Related to Figure 4 (A) Differentially expressed genes in PGCs and somatic cells at prim-5 stage. Significantly differentially expressed genes are in red. P adjusted < 0.1. (B) Reproducible ATAC peaks identified in function of the Irreproducible Discovery Rate (IDR) threshold. (C) Unsupervised Pearson correlation heatmap for developmental stages and cell types after selection of reproducible ATAC peaks. (D) Genome browser view of ATAC-seq tracks (magenta) of *wnt3* gene in PGCs and somatic cells across early development. Blue boxes highlight putative regulatory elements. Arrows show transcriptional directionality. (E) GO terms for genes associated with open chromatin regions upregulated in late somatic cells. (F) CpG base Pearson correlation shown as heatmap and histogram of CpG frequency.

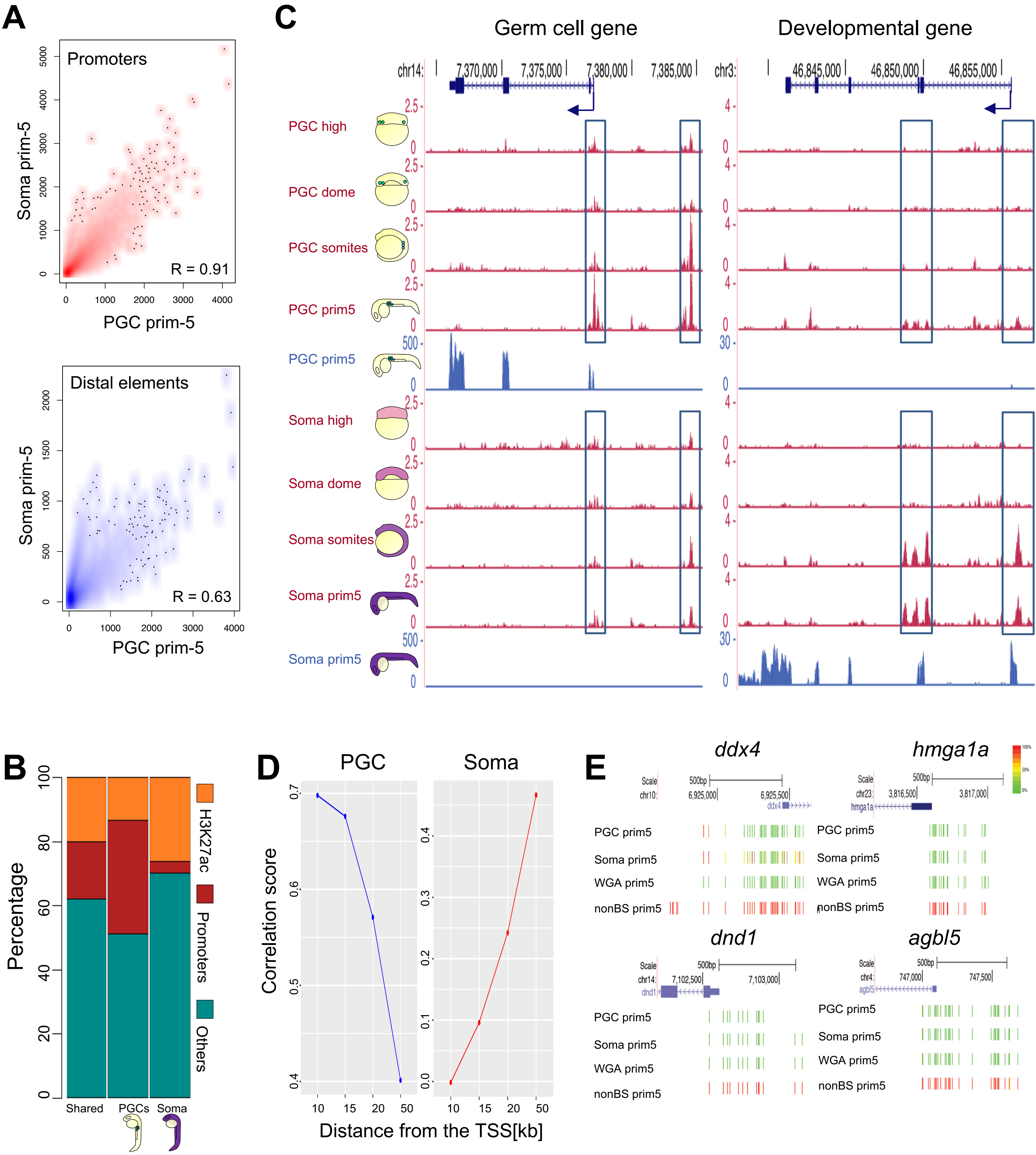

Supplementary Figure 5. PGCs do not open chromatin at regions identified as putative enhancers. Related to Figure 5 (A) Correlation scatter plots of ATAC peaks between PGCs and somatic cells for promoters and distal elements at prim-5 stage. (B) Percentage of ATAC peaks associated with H3K27ac and promoters in PGCs and somatic cells at prim-5 stage. (C) Genome browser view of open chromatin regions (ATAC-seq in magenta) and transcript levels (RNA-seq in blue) in PGCs and somatic cells. Blue boxes highlight putative regulatory elements and promoters. Arrows show transcriptional directionality. (D) Correlation score between chromatin accessibility and gene transcription in function of distance from the TSS. Correlation score is represented as the absolute ATAC log_2FC value associated to genes upregulated in PGCs (blue) or somatic cells (red) (E) Genome browser view of four representative promoters upregulated in late PGCs versus somatic cells. CpG methylation levels are reported as coloured bars.

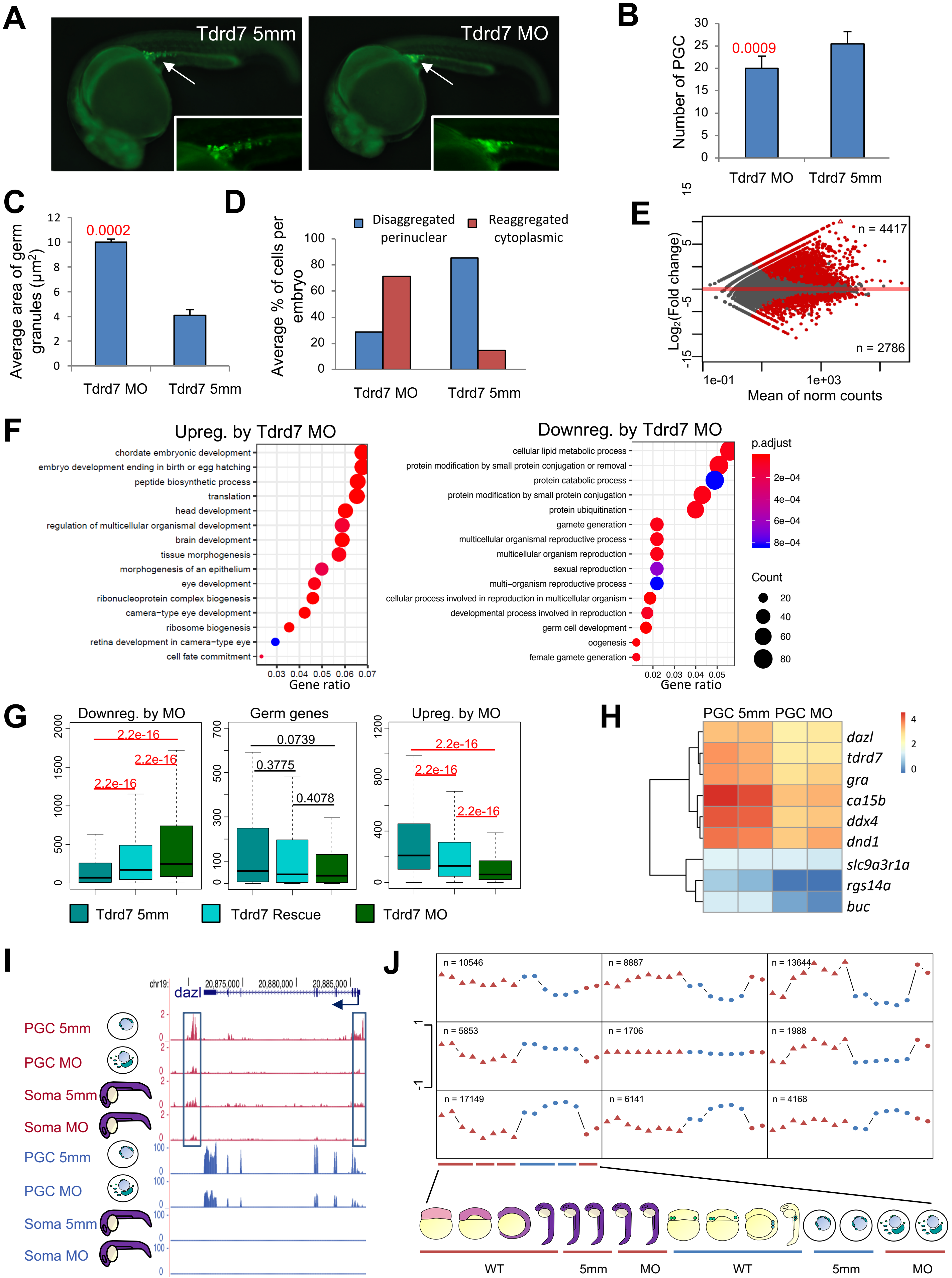
A
Tdrd7 5mm
Tdrd7 MO
B
Number of PGC
0.0009
Tdrd7 MO
Tdrd7 5mm
C
Average area of germ granules (μm²)
0.0002
Tdrd7 MO
Tdrd7 5mm
D
Disaggregated perinuclear
Reaggregated cytoplasmic
Average % of cells per embryo
Tdrd7 MO
Tdrd7 5mm
E
Log2(Fold change)
n = 4417
n = 2786
1e-01
1e+03
Mean of norm counts
F
Upreg. by Tdrd7 MO
chordate embryonic development
embryo development ending in birth or egg hatching
peptide biosynthetic process
translation
head development
regulation of multicellular organismal development
brain development
tissue morphogenesis
morphogenesis of an epithelium
eye development
ribonucleoprotein complex biogenesis
camera−type eye development
ribosome biogenesis
retina development in camera−type eye
cell fate commitment
Gene ratio
Downreg. by Tdrd7 MO
cellular lipid metabolic process
protein modification by small protein conjugation or removal
protein catabolic process
protein modification by small protein conjugation
protein ubiquitination
gamete generation
multicellular organismal reproductive process
multicellular organism reproduction
sexual reproduction
multi−organism reproductive process
cellular process involved in reproduction in multicellular organism
developmental process involved in reproduction
germ cell development
oogenesis
female gamete generation
Gene ratio
p.adjust
Count
G
Downreg. by MO
2.2e-16
Germ genes
0.0739
0.3775
0.4078
Upreg. by MO
Tdrd7 5mm
Tdrd7 Rescue
Tdrd7 MO
H
PGC 5mm PGC MO
dazl
tdrd7
gra
ca15b
ddx4
dnd1
slc9a3r1a
rgs14a
buc
I
chr19:
dazl
20,875,000
20,880,000
20,885,000
PGC 5mm
PGC MO
Soma 5mm
Soma MO
PGC 5mm
PGC MO
Soma 5mm
Soma MO
J
n = 10546
n = 8887
n = 13644
n = 5853
n = 1706
n = 1988
n = 17149
n = 6141
n = 4168
WT
5mm
MO
WT
5mm
MO

Supplementary Figure 6. **Tdrd7 knock-down impacts germ plasm morphology and transcriptome. Related to Figure 6** (A) Fluorescent microscopy of Buc-GFP-expressing embryos at prim-5 stage injected with *tdrd7* 5mm and *tdrd7* MO oligos ($8 < n < 14$). (B) Number of Buc-GFP-expressing cells per embryo at prim-5 stage. P-value against control is shown ($10 < n < 15$, $p < 0.05$). Red colour indicates significant difference based on Wilcoxon test. (C) Measurement of germ granule size. P-value against control is shown in red ($9 < n < 12$, $p < 0.05$). Based on Wilcoxon test. (D) Assessment of germ plasm phenotype shown as percentage of cells per embryo ($10 < n < 15$). (E) Differential expression analysis for genes upregulated and downregulated by Tdrd7 KD in PGCs at prim-5 stage. Significantly differentially expressed genes are in red. P adjusted < 0.1, $\log_2$FC < -1/> 1. (F) GO terms for genes upregulated and downregulated by Tdrd7 translational inhibition in PGCs. (G) Box plots of normalised RNA-seq counts (tpm) for selected gene classes. Numbers show p-values in red colour if < 0.005 or black if > 0.005 based on Wilcoxon test. (H) Gene expression heatmap for germ plasm-localised transcripts. Colour intensities indicate $\log_2$(tpm+1). (I) Genome browser view of ATAC profiles after morpholino injections. Open chromatin (ATAC-seq) is shown in magenta and normalised transcript levels (RNA-seq) are shown in blue. Arrows show transcriptional directionality. (J) Self-organising map of reproducible open chromatin regions. Wild type (WT) PGCs and somatic cells are represented as circles and triangles respectively. Tdrd7 KD PGCs are shown as red circles.
