# Supplementary material for: Germ cell differentiation requires Tdrd7-dependent chromatin and transcriptome reprogramming marked by germ plasm relocalization
Source: Dev Cell. 2021 Mar 8;56(5):641–656.e5. doi: 10.1016/j.devcel.2021.02.007 (PMC7957325; doi:10.1016/j.devcel.2021.02.007)
Supplement: Document S2. Article plus Supplemental information [file mmc8.pdf]

Developmental Cell

Article

# Germ cell differentiation requires Tdrd7-dependent chromatin and transcriptome reprogramming marked by germ plasm relocalization

## Graphical Abstract

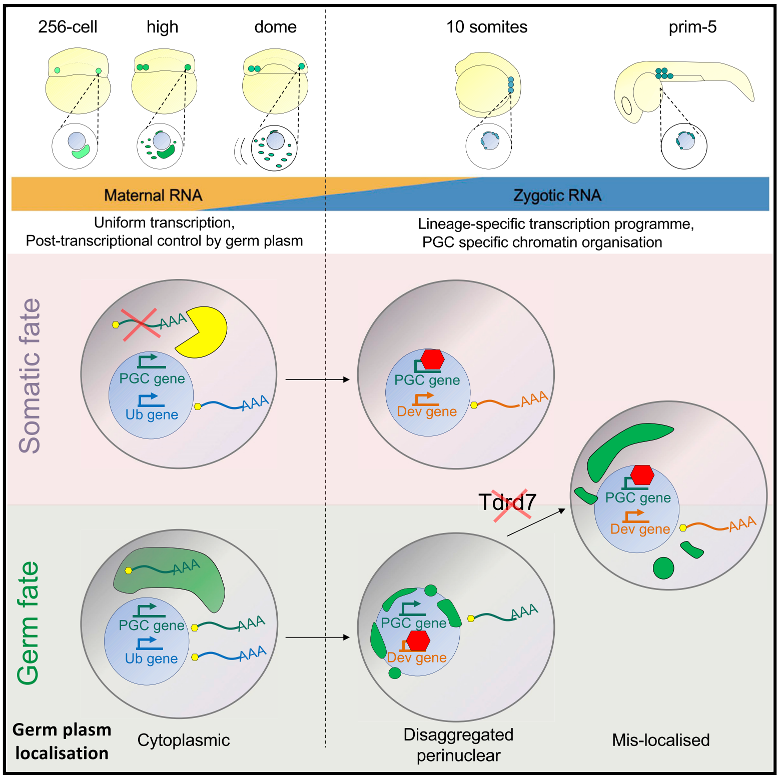


## Highlights

- No evidence for transcriptional activation delay in zebrafish PGCs
- Germ-plasm-associated post-transcriptional divergence during ZGA
- Epigenetic reprogramming marks onset of PGC migration
- Epigenetic reprogramming in PGCs relies on Tdrd7, coupled to germ plasm relocalization


## Authors

Fabio M. D'Orazio, Piotr J. Balwierz, Ada Jimenez González, ..., Bradley Cairns, Boris Lenhard, Ferenc Müller

## Correspondence

b.lenhard@imperial.ac.uk (B.L.), f.mueller@bham.ac.uk (F.M.)



## In brief

D'Orazio et al. show that zygotic genome activation in zebrafish primordial germ cells (PGCs) occurs in two phases. PGCs and somatic cells activate the genome concurrently, showing similar transcriptional and chromatin landscapes. Divergence occurs during PGC migration, when a PGC-specific chromatin landscape is coupled to Tdrd7a-dependent perinuclear germ plasm relocalization.


D'Orazio et al., 2021, Developmental Cell *56*, 641–656
March 8, 2021 © 2021 The Authors. Published by Elsevier Inc.
https://doi.org/10.1016/j.devcel.2021.02.007

CellPress

Developmental Cell

CellPress
OPEN ACCESS

Article

# Germ cell differentiation requires Tdrd7-dependent chromatin and transcriptome reprogramming marked by germ plasm relocalization

Fabio M. D'Orazio,[1,2,3] Piotr J. Balwierz,[1,2,3] Ada Jimenez González,[1] Yixuan Guo,[4] Benjamín Hernández-Rodríguez,[5] Lucy Wheatley,[1] Aleksandra Jasiulewicz,[1] Yavor Hadzhiev,[1] Juan M. Vaquerizas,[2,5] Bradley Cairns,[4] Boris Lenhard,[2,3,*] and Ferenc Müller[1,6,*]

[1]Institute of Cancer and Genomics Sciences, Birmingham Centre for Genome Biology, College of Medical and Dental Sciences, University of Birmingham, Birmingham, UK

[2]MRC London Institute of Medical Sciences and Faculty of Medicine, Imperial College, London, UK

[3]Institute of Clinical Sciences, Faculty of Medicine, Imperial College, London, UK

[4]Department of Oncological Sciences and Huntsman Cancer Institute, Howard Hughes Medical Institute, University of Utah School of Medicine, Salt Lake City, UT, USA

[5]Max Planck Institute for Molecular Biomedicine, Roentgenstrasse 20, Muenster, Germany

[6]Lead contact

*Correspondence: b.lenhard@imperial.ac.uk (B.L.), f.mueller@bham.ac.uk (F.M.)

https://doi.org/10.1016/j.devcel.2021.02.007

## SUMMARY

In many animal models, primordial germ cell (PGC) development depends on maternally deposited germ plasm, which prevents somatic cell fate. Here, we show that PGCs respond to regulatory information from the germ plasm in two distinct phases using two distinct mechanisms in zebrafish. We demonstrate that PGCs commence zygotic genome activation together with the somatic blastocysts with no demonstrable differences in transcriptional and chromatin opening. Unexpectedly, both PGC and somatic blastocysts activate germ-cell-specific genes, which are only stabilized in PGCs by cytoplasmic germ plasm determinants. Disaggregated perinuclear relocalization of germ plasm during PGC migration is regulated by the germ plasm determinant Tdrd7 and is coupled to dramatic divergence between PGC and somatic transcriptomes. This transcriptional divergence relies on PGC-specific *cis*-regulatory elements characterized by promoter-proximal distribution. We show that Tdrd7-dependent reconfiguration of chromatin accessibility is required for elaboration of PGC fate but not for PGC migration.

## INTRODUCTION

The germline ensures that parental genetic information is passed from one generation to the next. In sexually reproducing metazoans, the germ fate can be either oocyte-inherited (predetermined) (Eddy, 1975; Williamson and Lehmann, 1996) or zygotically triggered (induced) (Lawson et al., 1999; Ying et al., 2001). In mammals, germ cells are generated during gastrulation in response to extracellular signals from the surrounding embryonic cells (Ying et al., 2001). On the other hand, most non-mammalian model organisms, such as *C. elegans*, *D. melanogaster*, *X. laevis*, and *D. rerio*, require maternal transmission of germ-cell-specific factors (germ plasm) and their distribution into primordial germ cells (PGCs) (Eddy, 1975; Seydoux and Braun, 2006). The germ plasm has been shown to be sufficient and necessary to trigger the germ fate in zebrafish and frog (Gross-Thebing et al., 2017; Tada et al., 2012). The function of the germ plasm is to prevent somatic lineage differentiation of the host cells by at least two mechanisms. First, both vertebrates and invertebrates require germ plasm factors to regulate maternal RNA stability and translation (Charlesworth et al., 2006; Iguchi et al., 2006; Nakamura et al., 2004; Siddall et al., 2006; Krishnakumar et al., 2018; Wilhelm et al., 2003) that are cleared in the rest of the embryo as shown in zebrafish (Giraldez et al., 2006; Mishima et al., 2006). Thus, germ factors such as Nanos, Dazl, and Dead-end (Dnd) function in RNA processing pathways and are indispensable for PGC development in zebrafish and mouse (Köprunner et al., 2001; Suzuki et al., 2010). Second, germ plasm factors have been associated with block or delay of zygotic genome activation (ZGA) of the hosting cell resulting in somatic fate escape. In *C. elegans* and *D. melanogaster*, the germ plasm proteins PIE-1 and Pgc delay ZGA, allowing the disengagement between the germ and the somatic lines (Batchelder et al., 1999; Mello et al., 1996; Strome and Updike, 2015).

In contrast to the extensive, genome-wide DNA demethylation observed in migrating mammalian PGCs (Bender et al., 2004; Gkountela et al., 2015; Guo et al., 2015; Tang et al., 2015), epigenetic reprogramming has not been seen in zebrafish (Ortega-Recalde et al., 2019; Skvortsova et al., 2019). On the other hand,


This is an open access article under the CC BY license (http://creativecommons.org/licenses/by/4.0/).

epigenetic regulators maternally transmitted via the germ plasm have been implicated in germ fate acquisition in *C. elegans* (Gaydos et al., 2012; Rechtsteiner et al., 2010; Strome et al., 2014), suggesting that alternative mechanisms of germ-plasm-mediated transcriptional regulation may exist.

In this study, we aimed to characterize the function of the germ plasm during PGC formation. We hypothesized that the distinct localization patterns of the germ plasm before and during PGC migration may represent distinguishable cytoplasmic and nuclear-associated functions in PGC specification. We profiled transcriptome and epigenome of developing PGCs at high temporal resolution and discovered two distinct phases of PGC specification during zebrafish embryogenesis. We suggest that the early germ plasm does not influence transcription or chromatin landscape of the pre-migrating PGCs. However, the second phase requires chromatin reorganization, resulting in extensive transcriptional changes that coincide with the relocalization of germ granules from dispersed cytoplasmic to disaggregated perinuclear environment. Finally, by inhibiting the translation of Tudor domain 7a (Tdrd7a), which leads to disruption of germ plasm localization, we demonstrate its importance in defining PGC-specific open chromatin and transcriptional landscape.

## RESULTS

### Characterization of PGC transcriptome before and during migration

To investigate the role of germ granules, we set out to characterize the early germline development via extensive profiling of epigenetic and transcriptional features. We focused on the first day of zebrafish embryogenesis, when PGCs form and migrate to the genital ridge (Figure 1A). The Tg(Buc-GFP) line of *D. rerio* with fluorescently marked germ plasm (Riemer et al., 2015) was used to separate PGCs and non-fluorescent somatic cells by FACS (Figure S1A). Total transcriptome, open chromatin and DNA methylation were analyzed at multiple stages along zebrafish PGC development (Figure 1A). We first assessed transcriptome features associated with developmental stages and cell type and identified major changes coinciding with key events of development. Hierarchical clustering (Figure 1B) and principal-component analysis (PCA) (Figure 1C) demonstrated minimal transcriptome differences at and immediately after ZGA (high and dome stages) between replicates of germ-plasm-containing and somatic cells (Figure S1B; Table S1). Subsequent, gradual divergence between somatic and PGC transcriptomes was coincidental with migration of PGCs and disaggregated perinuclear localization of the germ plasm (10 somites stage), leading to a marked separation of steady-state transcriptome between PGCs and somatic cells by prim-5 stage.

In order to determine the transcriptional contribution to PGC development over time, we classified differentially expressed genes into four groups of temporal expression patterns using k-means clustering (Figures 1D and S1C). Within each cluster, genes with distinct biological functions could be identified, as highlighted by group-specific enrichment for gene ontology terms (Figure S1D). Genes in cluster 1 were upregulated in the somatic cells at 10 somites and prim-5 (late) stages over every other sample. These were associated with developmental processes, differentiation, and protein translation. Genes in cluster 2 were upregulated in late PGCs and included pathways of chromatin reorganization and DNA packaging. The low proliferative activity shown by PGCs was confirmed by downregulation of genes involved in cellular division (cluster 4), while cluster 3 was enriched for genes associated with germ fate.

Taken together, these results demonstrate successful isolation and genome-wide comparative characterization of PGCs at various developmental stages, which show stage- and cell-type-specific transcriptomic differences. Early germ cells have a similar transcriptional profile with the rest of the embryo, which gradually diverges as lineage specification proceeds.

### Zebrafish PGCs do not delay ZGA

Global transcriptomic analysis highlighted that PGCs and somatic cells are broadly similar during early development. This suggested that transcriptional activation may occur simultaneously in PGCs and somatic cells. Hence, we asked whether gene expression in PGCs is repressed when ZGA commences in all the blastomeres. We used a recently developed, *in vivo* transcription imaging tool (MOVIE) and 4D imaged the accumulation and localization of *microRNA-430* (*miR-430*) primary transcripts, which are the earliest known expressed genes during zebrafish embryogenesis (Giraldez et al., 2006; Hadzhiev et al., 2019). Upon injection of fluorescent morpholinos, we monitored *miR-430* expression in embryos in which germ plasm was labeled. Interestingly, *miR-430* expression was detectable in somatic as well as germ-plasm-containing cells already before the main wave of ZGA (Figures 2A, S2A, and S2B), indicating that germ plasm does not delay/inhibit early transcription. Also, *miR-430* expression faded around the onset of epiboly in both PGCs and somatic cells, confirming similar temporal transcriptional regulation.

To better understand the relation between ZGA and germ plasm, we studied the composition of PGC transcriptome by identifying genes differentially expressed over time using RNA sequencing (RNA-seq). We detected a drastic increase in gene upregulation over a short period of time. When germ plasm-carrying cells transition from 256-cell to high stage, 137 genes are significantly upregulated, while 1,929 genes are significantly upregulated between high to dome stages (false discovery rate [FDR]-adjusted p value (padj) $< 0.1$) (Table S2). Interestingly, of the 137 upregulated genes, 60 were predicted to be zygotically transcribed without maternal contribution (Figure 2B; STAR methods). We found several examples of transcript accumulation between 256-cell and high stages (Figure 2C). For instance, *irx7* transcription was undetectable at 256-cell stage while accumulated at high stage (Figure 2D). To validate our analysis, we compared our list of predicted zygotic genes with an independent RNA-seq dataset (White et al., 2017), showing a high degree of overlap (Figure S2C).

Interestingly, among the significantly upregulated genes in PGCs at high stage, transcripts associated with germ cell GOs and germ-plasm-localized transcripts such as *ddx4*, *dnd1*, *tdrd7a*, *gra*, and *dazl* were found to be upregulated from the previous stage (Figures 2B and S2D). This was unexpected as germ plasm markers are of maternal origin and were thought not to be transcribed until sphere stage (Blaser et al., 2005; Knaut et al., 2000; Weidinger et al., 1999).

The occurrence of ZGA in germ-plasm-carrying cells was further confirmed after performing a regression-based clustering of genes with similar expression profiles for three stages

CellPress
OPEN ACCESS

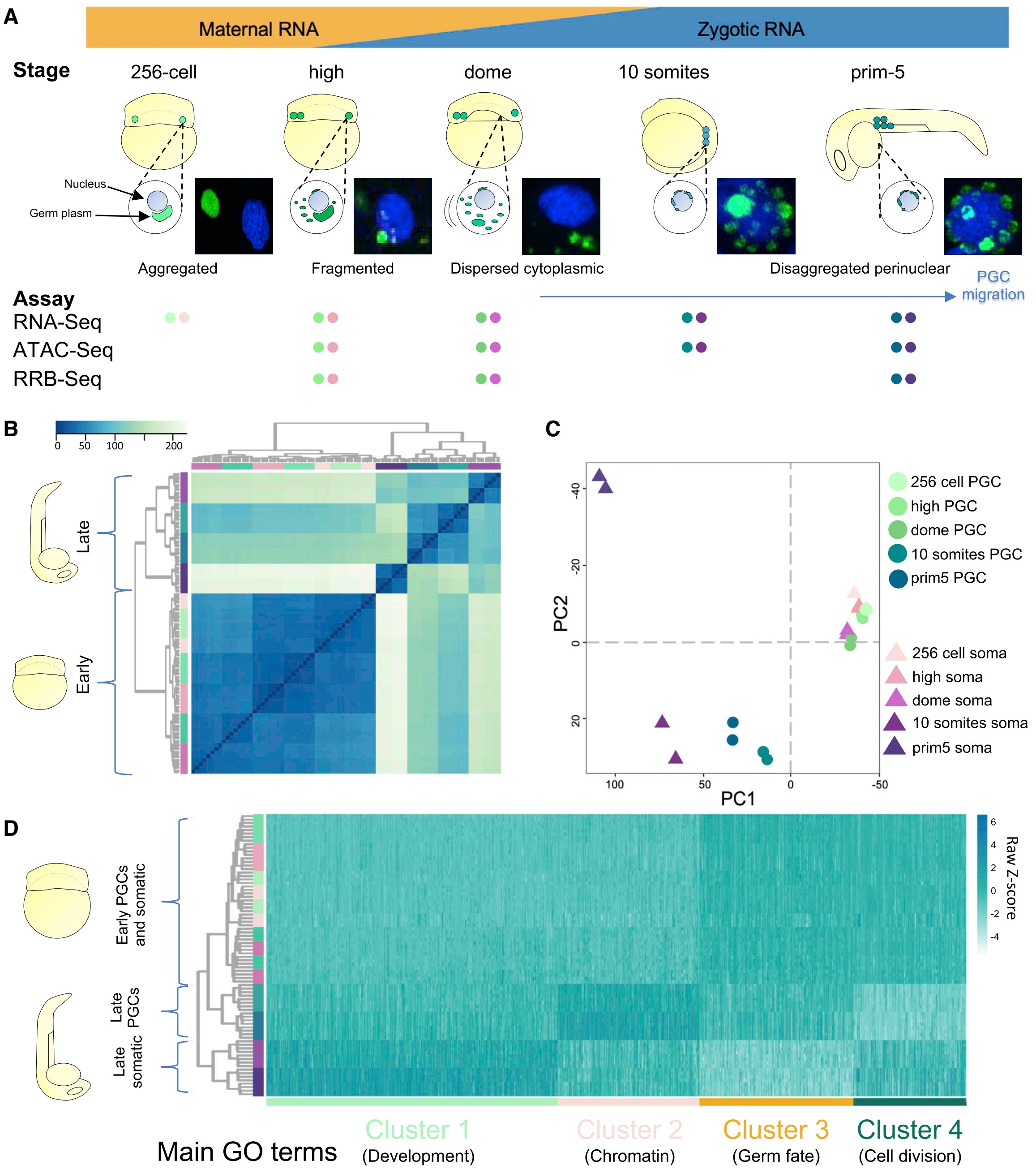


**Figure 1. Characterization of PGC transcriptome highlights early developmental similarities and late divergence between PGCs and somatic cells**

(A) Developmental stages used in the study are shown. Time points were selected according to various phases of germ plasm distribution/PGCs localization. Early stages span ZGA including the first wave at 256-cell stage. Fluorescent images show nuclei in blue (DAPI) and germ plasm in green (Buc-GFP). NGS assays performed for each time point are shown as colored dots; PGCs and somatic cells are in shades of green and purple, respectively. Data provided in biological duplicates unless stated otherwise.

(B and C) Unsupervised hierarchical clustering heatmap for Euclidean distance and two-dimensional PCA plot show developmental trends of PGC and somatic cell transcriptomes during development. PGCs and somatic cells are in shades of green and purple, respectively.

(D) Groups of differential gene expression reported as normalized transcript heatmap upon k-mean-based clustering over development and cell type.

spanning the first wave of ZGA (256-cell, high, and dome) in PGCs and somatic cells. We found two clusters of genes with ascending trends in both PGCs and somatic cells and one in which genes were upregulated in PGCs exclusively (Figure 2E).

Finally, we measured absolute RNA levels over time. After normalization for an internal RNA control (Jiang et al., 2011) (Figure S2E), we found a significant increase in transcript levels in both PGCs and somatic cells at high stage,

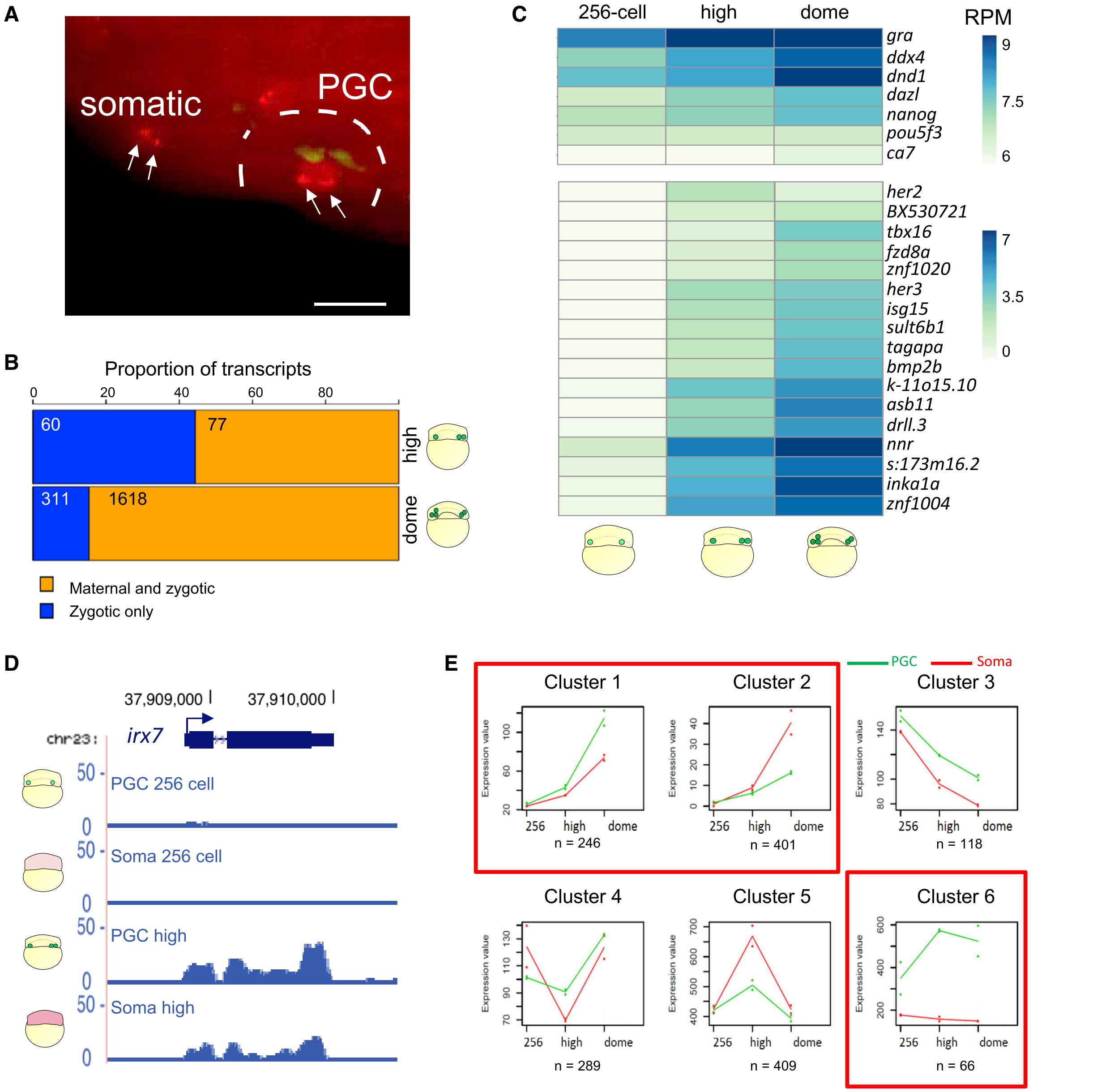


**Figure 2. PGCs do not delay the major wave of transcriptional activation**

(A) Maximum intensity projection of a multi-stack image showing *miR-430* transcription foci (arrows) detected by fluorescently tagged morpholinos (red) in cells marked by germ-plasm-localized GFP-Buc (green) and somatic cells at 512-cell stage. Scale bar, 30 μm. Number of embryos, n = 24.

(B) Proportion of zygotic and maternal/zygotic genes upregulated in the germ-plasm-carrying cells at high and dome stages.

(C) Expression heatmap of differentially regulated genes in germ-plasm-carrying cells. Scale bar represents scaled RPM. padj < 0.1.

(D) Genome browser view of normalized RNA-seq reads for *irx7* gene.

(E) Clusters of gene expression trends among three developmental stages in PGCs and somatic cells. Median profiles of gene expression values are plotted for each in green (PGCs) and red (somatic cells) and represent the read counts normalized by the DESeq2-calculated size factor. Red squares highlight clusters supporting transcriptional activation in PGCs.

confirming that active transcription is occurring in both cell types before high stage (Figure S2F).

Based on our results, we conclude that the germ plasm does not cause general transcriptional repression in zebrafish and that zebrafish PGCs do not delay ZGA as it has been seen in *C. elegans*, *D. melanogaster*, or *X. laevis*.

## Selective retention of zygotic transcripts explains transcriptome differences between germ-plasm-carrying cells and somatic cells at ZGA

The observation that germ-plasm-carrying cells do not delay transcriptional activation, yet they appear to carry *de-novo*-generated germ-cell-specific transcripts, prompted us to ask

whether differential transcription occurs between germ-plasm-carrying cells and somatic cells at ZGA. Hence, we performed differential gene expression analysis of isolated germ-plasm-carrying cells and somatic cells at each stage spanning ZGA period and found several differentially regulated genes (Figure 3A). This analysis revealed that, before ZGA, already 23 genes were differentially expressed between the two cell types (FDR < 0.1) (Table S2), confirming that maternal mRNAs are selectively retained in PGCs as shown previously (Eno et al., 2018; Gazdag et al., 2009; Gerovska and Araúzo-Bravo, 2016; Gorokhova et al., 2007; Levine et al., 2000; Rothschild et al., 2013). However, it is noteworthy that 12 out of 23 identified transcripts have not yet been associated with germ cell or PGC functions and are candidates for novel maternal germ plasm transcripts. The remaining 11 transcripts instead were either known germ plasm markers (*gra, tdrd7, rg514a, ca15b*, *dnd1*, and *dazl*) or were previously associated with germ cell development/survival (*hook2*, *tgfa*, *zswim5*, *b4galt6*, and *camk2g1*).

At high stage, we identified 142 genes significantly differently regulated between PGCs and somatic cells. Interestingly, the number of genes upregulated from one stage to the next was similar between PGCs and somatic cells during ZGA period (Figure S3A). In support, the relative abundance and the fold change of increased gene expression of transcripts upregulated from 256-cell to high stage were highly correlated between PGCs and somatic cells (Figures S3B and S3C), suggesting that the same set of genes was upregulated in both cell types.

To address whether retention of the germ plasm directly affects PGC-specific transcriptional activation, we monitored transcriptional activity using chromatin accessibility state at *cis*-regulatory elements as a proxy. We performed assay for transposase-accessible chromatin combined with sequencing (ATAC-seq) in PGCs and somatic cells. Open chromatin landscapes were analyzed globally and compared between PGCs and somatic cells at high and dome stages (start of main ZGA wave). This revealed a high degree of correlation between PGCs and somatic cells (Figure S3B). No distinguishable difference in chromatin accessibility for PGCs and somatic cells was observed on either promoters or distal elements of genes with differential expression between PGCs and somatic cells (Figure 3B). The germline gene *ddx4*, whose expression was shown by the RNA-seq analysis to increase from 256-cell to high stage only in PGCs, appeared to possess similar degree of chromatin accessibility at its promoter and *cis*-regulatory regions in both cell types (Figure 3C).

Taken together, these observations prompted us to hypothesize that, while zygotic transcriptional activation is broadly similar between somatic and germ plasm-carrying cells, differential gene expression is caused by post-transcriptional events.

While it was previously reported that maternal RNAs are selectively protected in PGCs from miRNA-dependent degradation (Mishima et al., 2006; Oulhen and Wessel, 2016), this mechanism has not yet been shown to occur on zygotically active germ cell genes. As the top-scoring upregulated genes in PGCs from 256-cell to high stage are known germ plasm markers, we hypothesized that these are not only deposited in the germ plasm but are also transcribed throughout the early embryo. To test this and to discriminate between maternally provided and zygotic germ plasm transcripts, we performed differential intron retention (IR) analysis (Middleton et al., 2017). As intron splicing is co-transcriptional (Merkhofer et al., 2014), newly transcribed RNAs are expected to show increased IR. We compared IR scores for the whole transcriptome before and after ZGA and observed increase of intron retention in both PGCs and somatic cells upon transcriptional activation (Figure S3E). Then, we focused on assessing IR in *de novo*, germ-cell-specific transcripts in somatic cells, and a significant increase in IR from the previous stage was observed when compared with random sampling of the dataset (Figure 3D).

To further validate this observation, we performed quantitative PCR (qPCR) on nuclear and cytoplasmic cell fractions before and after ZGA after removal of PGCs via fluorescence-activated cell sorting (FACS) (Table S3). Interestingly, we saw an increase in fold change expression for genes in the nuclear fraction but not in the cytoplasmic fraction, suggesting that germ-cell-related transcripts are transcribed by the somatic cells (Figure S3F).

To demonstrate this, we studied the localization of newly transcribed pre-mRNA within the embryo. Among the transcripts that were selectively upregulated in PGCs from 256-cell to high stage, *dazl* was one of the highest-scoring hits (Figure S3G). We therefore designed RNA probes targeting intronic sequences to visualize unprocessed, newly produced *dazl* pre-mRNA and carried out fluorescence *in situ* hybridization (FISH). Strikingly, *dazl* was seen to be actively transcribed in somatic cells, as demonstrated by staining of nuclear foci in the whole embryo at high stage (Figure 3E).

These results suggest that transcription of germ cell genes occurs throughout the embryo at ZGA. We have shown that there is no detectable transcriptional delay or differential transcription in germ-plasm-carrying cells when zygotic genome is activated. This was further supported by similar gene ontology (GO) terms resulting from genes upregulated during ZGA in PGCs and somatic cells (Figure S3H).

Selective protection of zygotic and maternal transcripts by the germ plasm may thus contribute to early PGC specification and onset of migration.

### PGCs gain specific transcriptomic and epigenetic features during migration

We next asked how transcriptome and chromatin states reflect the distinct ontogeny of PGCs and somatic cells during migration and further development. At dome stage, PGCs initiate independent movement and the germ plasm undergoes extensive morphological changes (Figure 1A) (Raz, 2003). This period is followed by the start of gastrulation and germ layer formation, with remarkable transformation of the whole embryo transcriptome upon lineage diversification (McKenna et al., 2016; Farrell et al., 2018; Raj et al., 2018). Accordingly, the number of genes differentially expressed between PGCs and somatic cells gradually increased over time (Figures 4A and S4A; Table S4). To assess whether transcriptional activity was occurring in both cell types, we looked at the number of genes upregulated from one stage to the next in PGCs and somatic cells separately. We noted that the increase in differential gene expression between PGCs and somatic cells was accompanied by an increase in differential gene expression over time (Figure 4B).

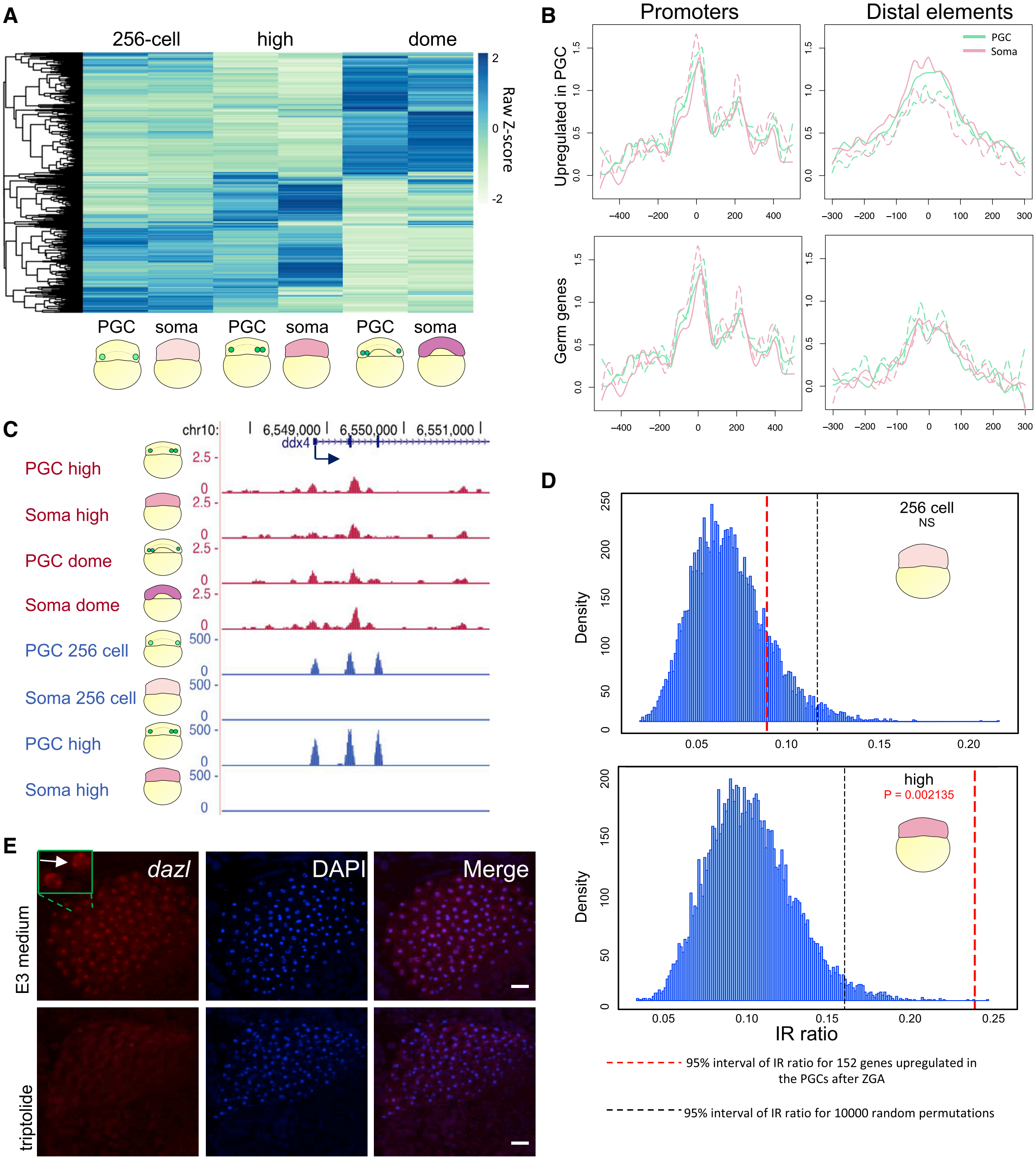


**Figure 3. Differential transcriptome between PGCs and somatic cells at early stages is not caused by differential transcription**

(A) Hierarchical clustering heatmap of differentially expressed genes between PGCs and somatic cells at indicated stages. Scale bar represents scaled RPM. padj < 0.1.

(B) Average chromatin accessibility signal at promoters and distal elements of PGC and somatic cells (dashed lines for replicates) for subgroups of genes at high stage. Promoters are aligned to transcription start site, while distal elements are aligned to peak center.

(C) Genome browser view of normalized ATAC-seq (magenta) and RNA-seq (blue) reads for PGC and somatic cells at stages spanning ZGA.

(D) Density of IR ratio before and after ZGA in the somatic cells for genes upregulated in the PGCs after ZGA. p value is calculated by t test. Statistical significance was calculated upon 10,000 random permutation whose density is shown before and after ZGA. Black and red dashed lines show 95% significance interval for the 10,000 permutations and the gene subgroup, respectively. NS, not significant.

(E) *In situ* hybridization for *dazl* pre-mRNA at high stage. Scale bar, 50 μm. Number of embryos, n = 7.

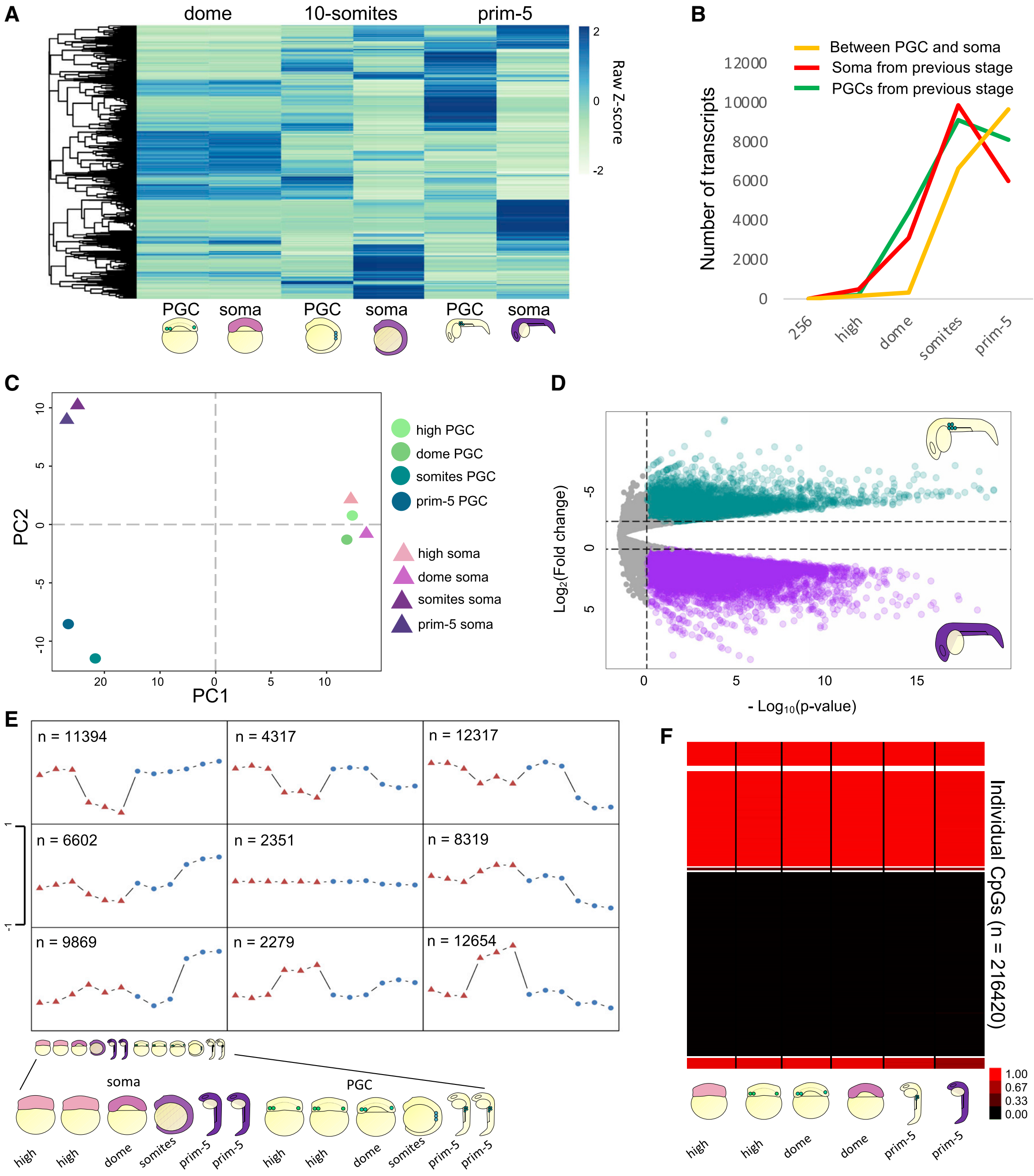


**Figure 4. Gradual acquisition of germ identity is accompanied by epigenetic changes**

(A) Hierarchical clustering heatmap of differentially expressed genes between PGCs and somatic cells at dome, 10-somites and prim-5 stages. Scale bar represents scaled RPM. padj < 0.1.

(B) Line-chart for gene counts. Upregulated genes from previous stage are in red (somatic) or green (PGCs). The orange line shows number of genes differentially expressed between PGCs and somatic cells at each stage.

(C) Two-dimensional PCA plot of ATAC-seq profiles.

(D) Volcano plot for regions of open chromatin between PGCs and somatic cells at prim-5 stage ($log_2$FC threshold = ±1, padj < 0.05).

(E) Self-organizing map of open chromatin regions. PGCs and somatic cells are shown as blue circles and red triangles, respectively. Schematic of embryos as in Figures 1A and 1B.

(F) Methylation status of identified CpGs in PGCs and somatic cells at indicated stages.

This observation prompted us to ask whether germ fate acquisition involves epigenetic and chromatin changes as previously described in murine PGCs. Therefore, we compared global chromatin accessibility in PGCs and somatic cells using ATAC-seq. After selection of ATAC peaks via irreproducible discovery rate (IDR) filtering (Zhang et al., 2017) (IDR < 0.05) (Figure 4B; Table S5), we compared global variability of chromatin accessibility among developmental stages and cell types (Figures 4C and S4C). In accordance with transcriptome results, early PGCs and somatic cells have similar open chromatin profiles and cluster by stage rather than by cell type. In contrast, after the gastrula period, a marked separation of PGC and somatic cell chromatin accessibility profiles was observed, revealing lineage-specific sites of open chromatin (Figure S4D).

Following differential chromatin accessibility analysis, we found 12,591 peaks more accessible in PGCs (logFC < −1, padj < 0.05) and 23,771 peaks in the somatic cells (logFC > 1, padj < 0.05) at prim-5 stage (Figure 4D). We then used self-organizing map (SOM) analysis on ATAC-seq data at different stages to identify patterns of *cis*-regulatory element accessibility via an unsupervised approach (Figure 4E). We identified 9 clusters. Of these, 9,869 sites were specific for late PGCs, while 12,654 sites were less accessible in PGCs compared with the late somatic cells. As expected, genes in proximity of somatic-specific ATAC peaks were associated with GO terms for embryonic morphogenesis, tissue formation, and development (Figure S4E).

To gain more insight into the epigenetic specification of PGCs, we profiled DNA methylome of pre- and migratory PGCs by performing reduced representation bisulfite sequencing (RRBS) (Murphy et al., 2018). In contrast to mammals (Guo et al., 2015; Hill et al., 2018), we found no extensive DNA methylation reprogramming of PGCs during these stages (Figures 4G and S4F). Analysis of differentially methylated CpGs between PGCs and somatic cells identified 3,825 significantly differentially methylated regions (Table S5) (only 1.77% of all recovered CpGs from all samples), revealing an overall highly similar methylation program, in accord with recent studies (Ortega-Recalde et al., 2019; Skvortsova et al., 2019). Based on this result, we concluded that DNA methylation dynamics and chromatin remodeling in zebrafish PGCs were uncoupled.

The finding that post-migratory PGCs show a specific chromatin accessibility landscape in contrast to the early germ-plasm-carrying cells suggests that the elaboration of germ fate occurs during PGC migration and coincides with the subcellular relocalization of the germ granules. In addition, differences between PGCs and somatic cells were more marked when chromatin accessibility was profiled in comparison with DNA methylation.

### PGC-specific open chromatin profile is enriched for promoter-proximal putative enhancers and depleted for promoter-distal, developmental putative enhancers

We then asked what epigenetic features of PGC genes in the genomic environmental context may contribute to transcription and elaboration of germ fate. In order to further dissect the chromatin accessibility state across the genome, we performed differential chromatin accessibility analysis (logFC > 1, padj < 0.05, fold enrichment > 4) and we focused on the distribution of differentially regulated ATAC peaks over genic elements. While most of the somatic ATAC peaks occurred at intergenic regions, PGC-specific ATAC peaks tended to coincide with a promoter (Figure 5A). In PGCs, 43% of the upregulated open regions were found within 1 kb from the transcriptional start site (TSS), while 11% was associated with introns. On the other hand, 41% of the upregulated regions in the somatic cells were found within introns, and only 6% was associated with promoters. Comparison between PGCs and somatic cells showed that the chromatin profiles were more dissimilar at non-promoter-associated open chromatin regions (Figure S5A). Then, we aimed to define putative somatic enhancer regions by intersecting the upregulated ATAC peaks with active enhancer histone mark (Bogdanovic et al., 2012). Out of all identified somatic-specific ATAC peaks (logFC > 1 and padj < 0.05), almost 30% were associated with H3K27ac histone marks. In contrast, less than 10% of PGC-specific ATAC peaks (logFC < −1) matched somatic H3K27ac peaks (Figure S5B). The functional relevance of cell-type-specific chromatin accessibility was estimated by GO analysis of genes associated with differential open chromatin regions away from promoters. As expected, somatic cells were enriched for open chromatin regions in proximity of genes for developmental and differentiation pathways, while PGC-specific ATAC peaks were found in proximity of genes for germ fate, cellular transport, and stem cell differentiation (Figures 5B and S5C).

As GO terms for both gene expression and chromatin accessibility pointed at similar pathways, we sought to verify whether accessible DNA regions would be predictive of transcriptional activity. We compared the fold change of gene expression between PGCs and somatic cells for a subset of transcripts characterized by significant differential open chromatin between the two cell types. Interestingly, we noted a significant correlation between cell-type-specific, promoter-associated ATAC peaks and transcriptional output in both PGCs and somatic cells (Figure 5C, left). On the other hand, the accessibility of distal elements was not predictable of transcription in PGCs, while a higher correlation between transcription and chromatin accessibility was observed in somatic cells (Figure 5C, right). This result suggests that transcriptional regulation in migratory PGCs is less dependent on distal elements. In support, we observed an inverse trend of correlation between open chromatin and gene expression relative to distance from the TSS for PGCs and somatic cells (Figure S5D). Moreover, the cumulative distribution of PGC/somatic-specific *cis*-regulatory elements relative to the closest TSS showed that PGC-specific *cis*-regulatory elements were more proximal to TSSs compared with somatic-specific ones (Figure 5D). These results indicate that regions of open chromatin predicted to drive gene expression from distance are less frequent in PGCs.

Next, we asked what epigenetic mechanisms may be involved in keeping putative distal enhancers closed in PGCs. Although little variation was seen in global DNA methylation profiles of PGCs and somatic cells at prim-5 stage (Figure 4E), we explored methylation dynamics in PGCs further by discriminating promoter and enhancer regions. When comparing methylation levels across stages and cell types, we observed minimal variation on promoter-overlapping CpGs. Out of 13 validated genes between PGCs and somatic cells, only *ddx4* showed differential DNA methylation on its promoter at prim-5 stage (Figure S5E). In contrast, while putative enhancer regions in somatic cells

CellPress
OPEN ACCESS

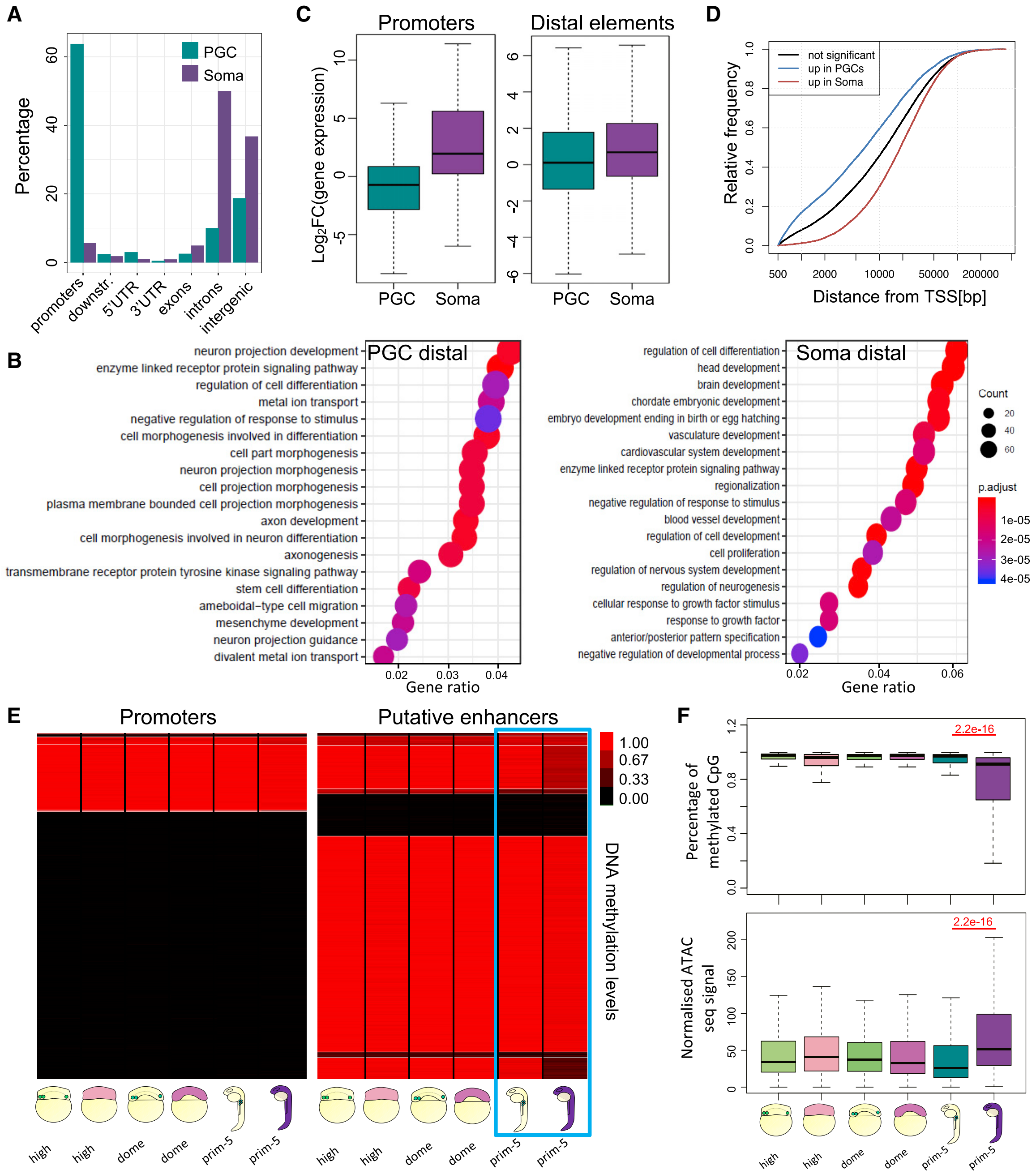


**Figure 5. PGCs do not open chromatin at regions identified as putative enhancers**

(A) Percentage of differentially accessible ATAC peaks in PGCs and somatic cells at prim-5 stage overlapping with gene features. Promoter regions include 1 kb up- and downstream of the TSS.

(B) Developmental processes GO analysis for genes associated with differentially accessible putative enhancers in PGCs and somatic cells.

(C) Fold change of gene expression for genes associated with ATAC peaks upregulated in PGCs or somatic cells. Fold changes represent gene upregulation in PGCs and somatic cells, respectively.

(D) Cumulative frequency of open chromatin elements in relation to distance from the closest TSS. padj < 0.05. Colors indicate elements near differentially expressed genes as indicated.

(E) Heatmap of average DNA methylation levels at promoters (left) and H3K4me1/H3K27ac-rich genomic sites (putative enhancers) in PGCs and somatic cells at high, dome, and prim-5 stages. Blue box highlights prim-5 stage.

(F) Quantification of methylated CpGs and chromatin accessibility at putative enhancer regions. p value in red was calculated by Wilcoxon test. Outliers are omitted.

experienced DNA demethylation during development, they were kept hypermethylated in PGCs at all the analyzed stages (Figure 5E; Table S5), similarly to that seen for a set of enhancer candidates in a recent study (Skvortsova et al., 2019). Interestingly, putative enhancers showed concomitant higher methylation and significant lower chromatin accessibility in PGCs compared with the somatic cells (Figure 5F). Next, we tested whether the observed methylation changes were coupled with changes in modulators of DNA methylation. We found upregulation in PGCs of *dnmt3bb.1* and *dnmt3bb.2* genes, while no significant change was detected for *dnmt1* gene. On the other hand, *tet2* expression was higher in the somatic cells than that in PGCs, suggesting that PGCs may have reduced activity of hydroxymethylation-mediated DNA demethylation catalysis (Breiling and Lyko, 2015; Hill et al., 2018). Based on these results, we propose that PGCs prevent somatic transcription program through PGC-specific block of chromatin opening at regulatory elements of somatic developmental genes.

### TDRD7a, a germ plasm-segregating protein, is required to maintain PGC-specific chromatin and transcriptome signature

Profiling of chromatin accessibility indicated that PGCs undergo chromatin reprogramming, coinciding with the transition of the germ granules from dispersed cytoplasmic to disaggregated perinuclear (onset of PGC migration) (Doitsidou et al., 2002; Houwing et al., 2007; Knaut et al., 2000; Roovers et al., 2018; Strasser et al., 2008; Updike et al., 2011; Weidinger et al., 2003). We hypothesized that the increasing divergence between PGC and somatic identities could be driven by mechanisms affecting transcriptome and chromatin in association with germ plasm relocalization pattern.

A known germ plasm marker is the protein Tudor domain 7 (Tdrd7). When translation of Tdrd7 is inhibited by morpholino (MO) interference, the germ plasm is incapable of fragmenting and forming dispersed granules (Figure 6A; Table S6) (Strasser et al., 2008). In order to test whether Tdrd7 and disaggregated perinuclear germ granules are required for the elaboration of PGC fate, we investigated the chromatin accessibility and transcriptome profiles of embryos, in which germ plasm localization was perturbed upon MO-mediated *tdrd7* knockdown (KD). Injected embryos did not show overall change in morphology, developmental delay, or mis-migration of the PGCs, although mild reduction in their number was observed (Figures S6A and S6B; Table S6). PGCs in Tdrd7-deficient embryos were characterized by reaggregation of germ plasm into large granules and reduced observable perinuclear distribution (Figures 6A, S6C, and S6D; Table S6). The germ plasm reaggregation phenotypes were substantially rescued by MO-insensitive *tdrd7* mRNA injection (Figure 6A).

Differential gene expression analysis of Tdrd7 KD and wild-type PGCs at prim-5 stage reported a remarkable effect upon loss of Tdrd7 (Figures 6B and S6E; Table S6). GO analysis indicated that reproduction and germ cell development-associated genes were significantly downregulated, while genes associated with developmental process and organogenesis were upregulated (Figure S6F).

Hence, in the absence of Tdrd7 and correct germ plasm distribution, PGC character was lost in favor of somatic fate. PCA analysis confirmed that loss of Tdrd7 in PGCs shifted their transcriptome profile toward a somatic-like transcriptome (Figures 6B and 6C). Detailed RNA-seq analysis highlighted that genes downregulated in PGCs upon Tdrd7 KD also include genes associated with pluripotency and zygotic genes for gametogenesis, while ubiquitously expressed housekeeping genes were unaffected (Figure 6C). The observed effects on subsets of differentially expressed genes were partially but significantly rescued by *tdrd7* mRNA injection indicating specificity of the KD (Figure S6G).

Maternally inherited germ plasm transcripts showed only slightly reduced levels in the Tdrd7-lacking PGCs as compared with control PGCs (Figure S6H). This could be caused by inhibition of late PGC-specific transcription upon *tdrd7* KD, while maternal mRNAs were preserved. Therefore, disruption of Tdrd7 function and germ plasm mis-localization leads to global deregulation of PGC transcriptional program and mild notable effect on germ plasm RNAs.

Next, we asked whether the causes of differential gene expression in *tdrd7* KD PGCs could be traceable to the chromatin state. Global analysis of open chromatin in *tdrd7* KD embryos revealed a marked effect by Tdrd7 loss in PGCs. Open chromatin of *tdrd7* KD PGCs showed somatic-like profiles, suggesting that the observed transcriptome phenotype was accompanied by chromatin changes (Figure 6D). Analysis of putative *cis*-regulatory element in proximity of misregulated genes in Tdrd7 morphant embryos showed a general tendency toward compaction at PGC-specific genes, while development- and morphogenesis-associated genes gained open chromatin peaks in comparison with control PGCs (Figure 6E).

Of note, PGC-specific genes, such as *dazl*, which are expressed zygotically as well as inherited maternally in the germ plasm, were shown to be associated with the closure of promoter and candidate enhancers despite only mild reduction in their RNA levels (Figure S6I).

To confirm the association between chromatin accessibility profile and reprogramming of PGCs toward the somatic fates, we have carried out a global analysis of open chromatin trends upon *tdrd7* KD and generated SOM classes of putative regulatory elements (Figure S6J). Unsupervised sample clustering confirmed distinct shift of chromatin states in PGCs toward somatic fate, when translation of *tdrd7* was inhibited and the germ granules were mis-localized. These results demonstrate transcription regulatory roles for the germ plasm determinant Tdrd7 and suggest the existence of crosstalk either direct or indirect between perinuclear disaggregated germ plasm and the nucleus during germ-cell-specific gene activation and fate decision.

## DISCUSSION

In this study, we have investigated the transcriptome, chromatin accessibility, and DNA methylation dynamics during early development of PGCs in a germ plasm-dependent vertebrate. We were able to describe two distinct roles for germ-cell-specific cytoplasmic granules. We linked these two roles with two distinct germ plasm subcellular distributions and distinguished an early and a late phase of germ fate differentiation in zebrafish. In contrast to many animal models, we demonstrated that germ

CellPress
OPEN ACCESS

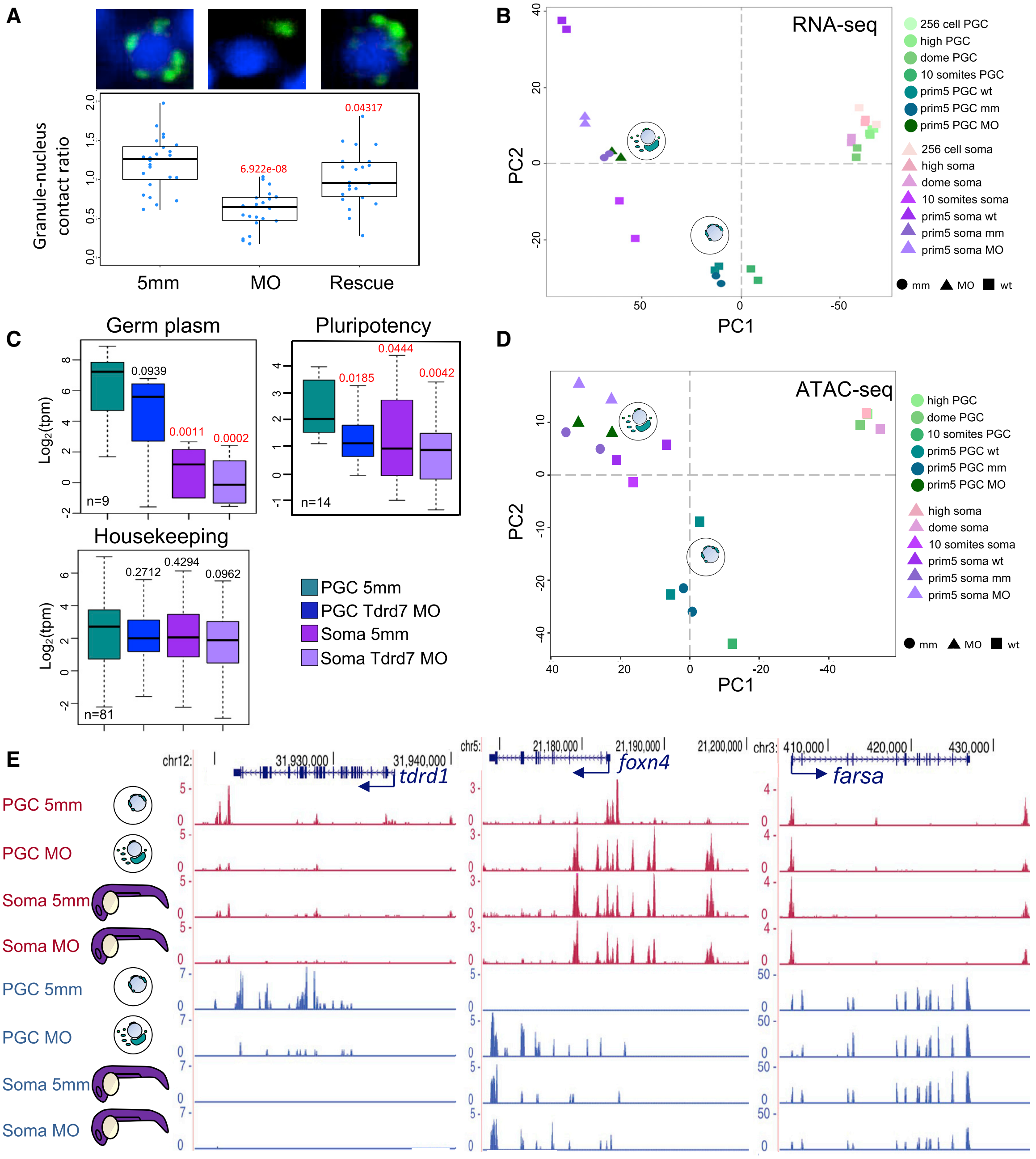


**Figure 6. Tdrd7 is required for maintaining PGC fate**

(A) Light-sheet images and phenotypic quantification of *tdrd7* KD in PGCs at prim-5 stage. Germ granule-nucleus contact ratio is calculated by contact length between each granule and nucleus divided by total granule surface area in a cell and shown as a single dot. Dot columns represent data from embryos (n = 6). Significance of p values against control is shown in red ($p < 0.05$, Wilcoxon test).

(B) Global transcriptional variance shown as PCA plot for wild-type and MO-injected PGCs and somatic cells.

(C) Boxplots reporting normalized transcript levels (tpm) for gene subsets in MO-injected PGCs and somatic cells. p values against control is shown. Red color indicates significance ($p < 0.05$, Wilcoxon test). Outliers are omitted.

(D) PCA analysis based on ATAC-seq peaks shows PGCs diverging toward somatic-like chromatin state.

(E) Genome browser view of ATAC-seq profiles after morpholino injections. Open chromatin (ATAC-seq) is shown in magenta and transcript levels (RNA-seq) are shown in blue. Arrows show transcription direction of genes indicated in blue.

plasm contributes to post-transcriptional regulation of both maternal and zygotic gene products, with no detectable effect on transcription during and after genome activation. Following commencement of PGC migration and relocalization of the germ plasm, PGC-specific chromatin accessibility is gained alongside PGC-specific transcription, which we show are Tdrd7-dependent and likely mediated by relocalization of the germ granules.

### ZGA is not delayed in zebrafish germ-plasm-carrying cells

Germ plasm factors block or delay ZGA by sequestering RNA polymerase II (RNA Poll II) or its co-factors in many organisms (Batchelder et al., 1999; Mello et al., 1996), thus preventing the commencement of developmental differentiation programs in the germline characteristic to developing somatic lineages. Also in mouse, in which PGCs are specified without maternal germ plasm (Lawson et al., 1999; Ohinata et al., 2009; Saitou and Yamaji, 2012), newly formed germ cells undergo transcriptional quiescence for a short period of time (Kurimoto et al., 2008). The mechanisms of transcription pausing are not fully understood; however, they involve inhibition of the elongation factor P-TEFb by Pgc and PIE-1 proteins in *D. melanogaster* and *C. elegans* (Hanyu-Nakamura et al., 2008; Mello et al., 1996) and sequestration of the general transcription factor TAF4 in *C. elegans* (Guven-Ozkan et al., 2008). In contrast, immunostaining of serine 2-phosphorylated RNA Poll II, showed localized nuclear foci in zebrafish germ-plasm-carrying cells as early as 256-cell stage (Knaut et al., 2000), suggesting that alternative mechanisms of germ fate differentiation may exist. In this study, we imaged transcription *in vivo* for the first time in PGCs and, together with transcriptome analysis, we demonstrate that the germ plasm does not delay the first wave of ZGA in zebrafish. These findings indicate striking plasticity in establishing the germ cell fate among clades and raise the prospect that transcriptional delay is not essential for PGC formation. Interestingly, massive transcriptional activation has been previously shown to cause double-strand break (DSB) in the embryonic germline (Butuči et al., 2015). Therefore, we speculate that zebrafish PGCs may experience a milder transition from a non-transcribing to a transcribing state while being less exposed to DNA damage.

### Why do PGCs need zygotic contribution to maternally deposited mRNAs?

We provided several lines of evidence to show that PGCs and somatic cells transcribe similar set of genes during blastula stages and that there is no overt difference in their chromatin states. Strikingly, we report that PGC-specific RNAs are also zygotically transcribed in somatic precursors, where their maternal counterparts are known to be promptly degraded by zygotic machineries (Giraldez et al., 2006; Mishima et al., 2006). Thus, it is unclear why somatic cells produce PGC-specific RNAs. This unexpected observation may be explained by chromatin organization of undifferentiated pluripotent stem cells, such as the blastomeres of the zebrafish embryo. Pluripotent cells display less compact chromatin organization than differentiating cells (Andrey and Mundlos, 2017) and are characterized by accessible chromatin with low but detectable activity of a broad range of genes. In contrast, differentiating cells are characterized by gradual divergence of accessibility of lineage and cell-type specific enhancers (Ladstätter and Tachibana, 2019; Lu et al., 2016; Perino and Veenstra, 2016). Therefore, it is feasible that during ZGA, zebrafish blastomeres carry a pluripotent ES-like chromatin and transcription state characterized by broad transcriptional capacity. This primordial state of chromatin organization likely lacks or only commences the formation of chromatin architecture characteristic of differentiating cells during development (Kaaij et al., 2018). It is conceivable that sophisticated, enhancer-dependent regulation has not yet been established, and gene expression occurs without lineage specificity, and as such, germ-cell-specific gene expression in somatic progenitors is tolerated. In this model, emphasis is on post-transcriptional control of gene expression, and germ plasm-mediated, selective stabilization of zygotic transcripts could be the primary source of divergence of transcriptome between PGCs and somatic cells. In support, several germ plasm factors are known to contribute to post-transcriptional and translational regulation. For example, the RNA-binding germ plasm component Dnd1 stabilizes maternal mRNAs selectively by restricting access of miRNAs responsible for clearing maternal mRNAs in somatic cells (Giraldez et al., 2006; Kedde et al., 2007). Likewise, the germ factors Nanos-1, -2, and -3 have been implicated in destabilization of RNAs and translational inhibition in the germ cells in combination with Pumilio and CCR4-NOT (Lee et al., 2017; Suzuki et al., 2012).

An additional question emerging from our finding is why zebrafish PGCs need zygotic transcription of maternally provided PGC-specific mRNAs. We speculate that such early activation of zygotic transcription is either redundant, similarly to the thousands of mRNAs, which are both present maternally and zygotically in somatic progenitors (Haberle et al., 2014; Harvey et al., 2013), or that the zygotic component is required to gradually take over and compensate for loss and/or dilution of maternal mRNA in dividing PGCs.

### A germ-plasm-mediated epigenetic reprogramming engages germ fate

PGCs initiate independent and active migratory movements by dome stage (Blaser et al., 2005; Bontems et al., 2009; Eno and Pelegri, 2016; Eno et al., 2018; Raz, 2003; Yoon et al., 1997), indicating commencement of germ-line-specific cues. Early migratory movements are triggered by Dnd-mediated loss of cell adhesion (Blaser et al., 2005) as well as the interaction between the C-X-C chemokine receptor type 4b (Cxcr4b) on PGC surface and the ligand Cxcl12a (Doitsidou et al., 2002; Knaut et al., 2003). Additionally, translational inhibition of *nanos* and *oskar* mediated by the germ plasm factors Bruno and CUP is required during germ cell formation in *D. melanogaster* (Nakamura et al., 2004; Wilhelm et al., 2003), while the RNA-binding protein DAZL inhibits translation of several mRNAs involved in pluripotency, somatic differentiation, and apoptosis in mouse PGCs (Chen et al., 2014). Therefore, the germ plasm is actively involved in post-transcriptional regulation of embryonic transcripts, providing germ-plasm-carrying cells with unique means, likely required for triggering migration and providing foundation for downstream molecular cues, which will initiate the second phase of PGC development.

CellPress
OPEN ACCESS

We have demonstrated that chromatin accessibility reprogramming occurs in zebrafish PGCs after migration has started. We have reported a unique chromatin accessibility pattern in PGCs, which is coupled to germ plasm relocalization. Interference with germ plasm localization upon inhibition of its regulator Tdrd7 leads to loss of PGC-specific *cis*-regulatory element accessibility patterns and PGC fate. Intriguingly, these chromatin- and transcriptome-compromised PGCs correctly migrate but experience somatic-like gene regulation. Notably, DNA methylation analysis demonstrate that even in the absence of global DNA demethylation (Macleod et al., 1999; Bogdanović et al., 2016; Skvortsova et al., 2019), local, differential methylation between PGCs and somatic cells is observed at regulatory sites. However, in contrast to mammalian PGCs, where DNA methylation is almost completely erased genome wide (Guo et al., 2015; Hill et al., 2018), we observe an inverse trend in zebrafish, where hypermethylation is found on somatic putative enhancers, suggesting remarkably different mechanism for epigenetic regulation of the germline among vertebrates. This was further corroborated with open chromatin analysis genome wide, which showed lack of opening somatic enhancers in PGCs. Interestingly, although our study led to the identification of PGC-specific putative regulatory elements, we found low correlation between TSS-distal ATAC peaks and transcription, while higher correlation between chromatin accessibility and transcription was detected for TSS-proximal *cis*-acting elements. This finding, in line with previous reports in humans (Guo et al., 2017), suggests that transcriptional activation in PGCs is achieved by short-range interactions. Nevertheless, additional studies focusing on the DNA interactions, topological associated domains formation, and spatial organization of the chromatin in the developing germline will contribute to better understand the mechanisms of acquisition and maintenance of totipotency.

### Importance of germ plasm components and subcellular organization

In accordance with previous studies, our results confirm that removal of individual germ plasm components is sufficient to trigger somatic differentiation in PGCs (Gross-Thebing et al., 2017; Lee et al., 2017). Interestingly, Tdrd7-depleted PGCs preserve germ granules and germ factors and correctly reach the genital ridge (Hosokawa et al., 2007; Strasser et al., 2008), suggesting that pathways upstream migratory movements are unaffected. We are unable to answer whether the correct migration of PGCs in the Tdrd7 morphant indicates loss of fertility, or partial loss of function, maternal effect compensation of zygotic loss of gene activities, or redundancy of Tdrd7 by a yet unknown mechanism. Notably, PGCs in Tdrd12 mutants were reported to migrate correctly yet were shown to be infertile (Dai et al., 2017). However, despite this mild phenotype, we report a remarkable somatic-like chromatin accessibility and consequent transcriptional reprogramming. Hence, we speculate that lack of Tdrd7 and incorrect germ granule localization are sufficient to diverge the fate of the embryonic germline and induce activation of somatic differentiation pathways.

Tdrd7 is known to interact with Piwi, a piRNAs processor (Huang et al., 2011). Piwi-mediated piRNAs processing has been associated with epigenetic changes in both the somatic and the germline (Houwing et al., 2007); therefore, it is tempting to speculate about a role for piRNA pathways. For example, piRNAs are known to control transposon silencing via H3K9me3 and, in general, to regulate chromatin state on piRNA-target regions (Huang et al., 2011; Sienski et al., 2012). Interestingly, it has been recently reported that germ granules protect germline transcripts from piRNA-mediated silencing, regulating the pace of release from the cytoplasm to the nucleus (Ouyang et al., 2019).

In conclusion, we suggest that disaggregated perinuclear localization of the germ plasm and Tdrd7 are involved in chromatin reprogramming of gonadal PGCs during somitogenesis. Our discoveries have implications in the understanding of pluripotent fate acquisition and the functional relationship between subcellular aggregates with epigenetic and chromatin reprogramming.

## STAR★METHODS

Detailed methods are provided in the online version of this paper and include the following:

- KEY RESOURCES TABLE
- RESOURCE AVAILABILITY
  - Lead contact
  - Materials availability
  - Data and code availability
- EXPERIMENTAL MODEL AND SUBJECT DETAILS
  - Zebrafish
- METHOD DETAILS
  - Microinjections of zebrafish embryos
  - Production of *tdrd7* RNA
  - Transcription block
  - PGC preparation for FACS
  - Fluorescent in-situ hybridization
  - Imaging
  - Nuclear-cytoplasmic fractionation and qPCR
  - Reduced representation bisulfite sequencing (RRBS-Seq)
  - Patch bisulfite PCR
  - ATAC-seq library preparation and sequencing
  - RNA-seq library preparation and sequencing
- QUANTIFICATION AND STATISTICAL ANALYSIS
  - Image processing and analysis
  - ATAC-seq analysis
  - Enhancer calling in somatic cells and PGCs
  - Principal component analysis and self organising map clustering
  - Bioinformatic analysis for RRBS
  - Bioinformatic analysis for Patch bisulfite PCR
  - RNA-seq analysis
  - Statistics

## SUPPLEMENTAL INFORMATION

Supplemental Information can be found online at https://doi.org/10.1016/j.devcel.2021.02.007.

## ACKNOWLEDGMENTS

This work was supported by the Wellcome Trust Investigator award (106955/Z/15/Z) H2O20 Zencode-ITN and BBSRC (BB/L010488/1) to F.M. and B.L. as

well as by a HFSP program grant to F.M. and B.C. and NEURAM FET H2020 project of the European Commission to F.M. We thank Brian Dalley for sequencing expertise, K.T. Varley for helping us adapt Patch bisulfite PCR, and the HCI high-throughput genomics core, flow cytometry core, fish facility at the University of Utah. Financial support was received from the Howard Hughes Medical Institute and the Huntsman Cancer Institute core facilities (CA042014). We thank Erez Raz for *tdrd7* expression construct and Katsiaryna Tarbashevicz at the ZMBE, Muenster for advice and comments on the manuscript. We thank Roland Dosch at the UMC, Göttingen for providing Tg(Buc-GFP) line and the BMSU facility at the University of Birmingham for zebrafish maintenance. We thank the Tech Hub core at the University of Birmingham for flow cytometry and genomics support.

## AUTHOR CONTRIBUTIONS

F.M.D. and F.M. conceived the study and wrote the manuscript. All the authors critically revised the manuscript. All experiments were performed by F.M.D. except for the following: A.J.G. carried out mRNA injection rescue experiments in embryos for sorting PGCs and RNA preparation. F.M.D., A.J.G., A.J., and Y.H. carried out light-sheet microscopy imaging and image processing. F.M.D. and L.W. performed confocal microscopy imaging and image processing of morpholino-injected Buc-GFP embryos. Y.G. generated RRBS libraries from isolated genomic DNA. P.J.B. mapped and analyzed ATAC-seq data with F.M.D. and with advice by B.L.; RNA-seq analysis was performed by B.H.-R. with J.M.V. and F.M.D.; RRB-seq analysis was designed and performed by Y.G. and B.C.

## DECLARATION OF INTERESTS

The authors declare no competing interests.

Received: January 21, 2020
Revised: October 25, 2020
Accepted: February 3, 2021
Published: March 1, 2021

## REFERENCES

Andrey, G., and Mundlos, S. (2017). The three-dimensional genome: regulating gene expression during pluripotency and development. Development *144*, 3646–3658.

Batchelder, C., Dunn, M.A., Choy, B., Suh, Y., Cassie, C., Shim, E.Y., Shin, T.H., Mello, C., Seydoux, G., and Blackwell, T.K. (1999). Transcriptional repression by the Caenorhabditis elegans germ-line protein PIE-1. Genes Dev. *13*, 202–212.

Bender, L.B., Cao, R., Zhang, Y., and Strome, S. (2004). The MES-2/MES-3/MES-6 complex and regulation of histone H3 methylation in C. elegans. Curr. Biol. *14*, 1639–1643.

Blaser, H., Eisenbeiss, S., Neumann, M., Reichman-Fried, M., Thisse, B., Thisse, C., and Raz, E. (2005). Transition from non-motile behaviour to directed migration during early PGC development in zebrafish. J. Cell Sci. *118*, 4027–4038.

Bogdanovic, O., Fernandez-Miñán, A., Tena, J.J., de la Calle-Mustienes, E., Hidalgo, C., van Kruysbergen, I., van Heeringen, S.J., Veenstra, G.J., and Gómez-Skarmeta, J.L. (2012). Dynamics of enhancer chromatin signatures mark the transition from pluripotency to cell specification during embryogenesis. Genome Res. *22*, 2043–2053.

Bogdanović, O., Smits, A.H., de la Calle Mustienes, E., Tena, J.J., Ford, E., Williams, R., Senanayake, U., Schultz, M.D., Hontelez, S., van Kruijsbergen, I., et al. (2016). Active DNA demethylation at enhancers during the vertebrate phylotypic period. Nat. Genet. *48*, 417–426.

Bontems, F., Stein, A., Marlow, F., Lyautey, J., Gupta, T., Mullins, M.C., and Dosch, R. (2009). Bucky ball organizes germ plasm assembly in zebrafish. Curr. Biol. *19*, 414–422.

Breiling, A., and Lyko, F. (2015). Epigenetic regulatory functions of DNA modifications: 5-methylcytosine and beyond. Epigenet. Chromatin *8*, 24.

Buenrostro, J.D., Giresi, P.G., Zaba, L.C., Chang, H.Y., and Greenleaf, W.J. (2013). Transposition of native chromatin for fast and sensitive epigenomic profiling of open chromatin, DNA-binding proteins and nucleosome position. Nat. Methods *10*, 1213–1218.

Butuči, M., Wong, M.M., and Michael, W.M. (2015). Trouble in transitioning: activation of zygotic transcription can lead to DNA breakage and genome instability. Worm *4*, e1115946.

Charlesworth, A., Wilczynska, A., Thampi, P., Cox, L.L., and MacNicol, A.M. (2006). Musashi regulates the temporal order of mRNA translation during Xenopus oocyte maturation. EMBO J. *25*, 2792–2801.

Chen, H.H., Welling, M., Bloch, D.B., Muñoz, J., Mientjes, E., Chen, X., Tramp, C., Wu, J., Yabuuchi, A., Chou, Y.-F., et al. (2014). DAZL limits pluripotency, differentiation, and apoptosis in developing primordial germ cells. Stem Cell Rep. *3*, 892–904.

Dai, X., Shu, Y., Lou, Q., Tian, Q., Zhai, G., Song, J., Lu, S., Yu, H., He, J., and Yin, Z. (2017). Tdrd12 is essential for germ cell development and maintenance in zebrafish. Int. J. Mol. Sci. *18*, 1127.

Doitsidou, M., Reichman-Fried, M., Stebler, J., Köprunner, M., Dörries, J., Meyer, D., Esguerra, C.V., Leung, T., and Raz, E. (2002). Guidance of primordial germ cell migration by the chemokine SDF-1. Cell *111*, 647–659.

Eddy, E.M. (1975). Germ plasm and the differentiation of the germ cell line. Int. Rev. Cytol. *43*, 229–280.

Eno, C., Gomez, T., Slusarski, D.C., and Pelegri, F. (2018). Slow calcium waves mediate furrow microtubule reorganization and germ plasm compaction in the early zebrafish embryo. Development *145*, dev156604.

Eno, C., and Pelegri, F. (2016). Germ cell determinant transmission, segregation, and function in the zebrafish embryo. In Insights from Animal Reproduction, R. Payan Carreira, ed. (InTech).

Farrell, J.A., Wang, Y., Riesenfeld, S.J., Shekhar, K., Regev, A., and Schier, A.F. (2018). Single-cell reconstruction of developmental trajectories during zebrafish embryogenesis. Science *360*, eaar3131.

Gaydos, L.J., Rechtsteiner, A., Egelhofer, T.A., Carroll, C.R., and Strome, S. (2012). Antagonism between MES-4 and polycomb repressive complex 2 promotes appropriate gene expression in C. elegans germ cells. Cell Rep. *2*, 1169–1177.

Gazdag, E., Santenard, A., Ziegler-Birling, C., Altobelli, G., Poch, O., Tora, L., and Torres-Padilla, M.E. (2009). TBP2 is essential for germ cell development by regulating transcription and chromatin condensation in the oocyte. Genes Dev. *23*, 2210–2223.

Gerovska, D., and Araúzo-Bravo, M.J. (2016). Does mouse embryo primordial germ cell activation start before implantation as suggested by single-cell transcriptomics dynamics? Mol. Hum. Reprod. *22*, 208–225.

Giraldez, A.J., Mishima, Y., Rihel, J., Grocock, R.J., Van Dongen, S., Inoue, K., Enright, A.J., and Schier, A.F. (2006). Zebrafish MiR-430 promotes deadenylation and clearance of maternal mRNAs. Science *312*, 75–79.

Gkountela, S., Zhang, K.X., Shafiq, TiashaA., Liao, W.-W., Hargan-Calvopiña, J., Chen, P.-Y., and Clark, A.T. (2015). DNA demethylation dynamics in the human prenatal germline. Cell *161*, 1425–1436.

Gorokhova, S., Bibert, S., Geering, K., and Heintz, N. (2007). A novel family of transmembrane proteins interacting with beta subunits of the Na,K-ATPase. Hum. Mol. Genet. *16*, 2394–2410.

Gross-Thebing, T., Yigit, S., Pfeiffer, J., Reichman-Fried, M., Bandemer, J., Ruckert, C., Rathmer, C., Goudarzi, M., Stehling, M., Tarbashevich, K., et al. (2017). The vertebrate protein dead end maintains primordial germ cell fate by inhibiting somatic differentiation. Dev. Cell *43*, 704–715.e5.

Guo, H., Hu, B., Yan, L., Yong, J., Wu, Y., Gao, Y., Guo, F., Hou, Y., Fan, X., Dong, J., et al. (2017). DNA methylation and chromatin accessibility profiling of mouse and human fetal germ cells. Cell Res *27*, 165–183.

Guo, F., Yan, L., Guo, H., Li, L., Hu, B., Zhao, Y., Yong, J., Hu, Y., Wang, X., Wei, Y., et al. (2015). The transcriptome and DNA Methylome landscapes of human primordial germ cells. Cell *161*, 1437–1452.

Guven-Ozkan, T., Nishi, Y., Robertson, S.M., and Lin, R. (2008). Global transcriptional repression in C. elegans germline precursors by regulated sequestration of TAF-4. Cell *135*, 149–160.

CellPress
OPEN ACCESS

Haberle, V., Li, N., Hadzhiev, Y., Plessy, C., Previti, C., Nepal, C., Gehrig, J., Dong, X., Akalin, A., Suzuki, A.M., et al. (2014). Two independent transcription initiation codes overlap on vertebrate core promoters. Nature *507*, 381–385.

Hadzhiev, Y., Qureshi, H.K., Wheatley, L., Cooper, L., Jasiulewicz, A., Van Nguyen, H., Wragg, J.W., Poovathumkadavil, D., Conic, S., Bajan, S., et al. (2019). A cell cycle-coordinated polymerase II transcription compartment encompasses gene expression before global genome activation. Nat. Commun. *10*, 691.

Hanyu-Nakamura, K., Sonobe-Nojima, H., Tanigawa, A., Lasko, P., and Nakamura, A. (2008). Drosophila Pgc protein inhibits P-TEFb recruitment to chromatin in primordial germ cells. Nature *451*, 730–733.

Harvey, S.A., Sealy, I., Kettleborough, R., Fenyes, F., White, R., Stemple, D., and Smith, J.C. (2013). Identification of the zebrafish maternal and paternal transcriptomes. Development *140*, 2703–2710.

Hill, P.W.S., Leitch, H.G., Requena, C.E., Sun, Z., Amouroux, R., Roman-Trufero, M., Borkowska, M., Terragni, J., Vaisvila, R., Linnett, S., et al. (2018). Epigenetic reprogramming enables the transition from primordial germ cell to gonocyte. Nature *555*, 392–396.

Hosokawa, M., Shoji, M., Kitamura, K., Tanaka, T., Noce, T., Chuma, S., and Nakatsuji, N. (2007). Tudor-related proteins TDRD1/MTR-1, TDRD6 and TDRD7/TRAP: domain composition, intracellular localization, and function in male germ cells in mice. Dev. Biol. *301*, 38–52.

Houwing, S., Kamminga, L.M., Berezikov, E., Cronembold, D., Girard, A., van den Elst, H., Filippov, D.V., Blaser, H., Raz, E., Moens, C.B., et al. (2007). A role for Piwi and piRNAs in germ cell maintenance and transposon silencing in zebrafish. Cell *129*, 69–82.

Huang, H.Y., Houwing, S., Kaaij, L.J., Meppelink, A., Redl, S., Gauci, S., Vos, H., Draper, B.W., Moens, C.B., Burgering, B.M., et al. (2011). Tdrd1 acts as a molecular scaffold for Piwi proteins and piRNA targets in zebrafish. EMBO J. *30*, 3298–3308.

Iguchi, N., Tobias, J.W., and Hecht, N.B. (2006). Expression profiling reveals meiotic male germ cell mRNAs that are translationally up- and down-regulated. Proc. Natl. Acad. Sci. USA *103*, 7712–7717.

Jiang, L., Schlesinger, F., Davis, C.A., Zhang, Y., Li, R., Salit, M., Gingeras, T.R., and Oliver, B. (2011). Synthetic spike-in standards for RNA-seq experiments. Genome Res. *21*, 1543–1551.

Kaaij, L.J.T., van der Weide, R.H., Ketting, R.F., and de Wit, E. (2018). Systemic loss and gain of chromatin architecture throughout zebrafish development. Cell Rep *24*, 1–10.e4.

Kedde, M., Strasser, M.J., Boldajipour, B., Oude Vrielink, J.A., Slanchev, K., le Sage, C., Nagel, R., Voorhoeve, P.M., van Duijse, J., Ørom, U.A., et al. (2007). RNA-binding protein Dnd1 inhibits microRNA access to target mRNA. Cell *131*, 1273–1286.

Knaut, H., Pelegri, F., Bohmann, K., Schwarz, H., and Nüsslein-Volhard, C. (2000). Zebrafish vasa RNA but not its protein is a component of the germ plasm and segregates asymmetrically before germline specification. J. Cell Biol. *149*, 875–888.

Knaut, H., Werz, C., Geisler, R., and Nüsslein-Volhard, C.; Tübingen 2000 Screen Consortium (2003). A zebrafish homologue of the chemokine receptor Cxcr4 is a germ-cell guidance receptor. Nature *421*, 279–282.

Köprunner, M., Thisse, C., Thisse, B., and Raz, E. (2001). A zebrafish nanos-related gene is essential for the development of primordial germ cells. Genes Dev. *15*, 2877–2885.

Krishnakumar, P., Riemer, S., Perera, R., Lingner, T., Goloborodko, A., Khalifa, H., Bontems, F., Kaufholz, F., El-Brolosy, M.A., and Dosch, R. (2018). Functional equivalence of germ plasm organizers. PLoS Genet. *14*, e1007696.

Kurimoto, K., Yabuta, Y., Ohinata, Y., Shigeta, M., Yamanaka, K., and Saitou, M. (2008). Complex genome-wide transcription dynamics orchestrated by Blimp1 for the specification of the germ cell lineage in mice. Genes Dev. *22*, 1617–1635.

Ladstätter, S., and Tachibana, K. (2019). Genomic insights into chromatin reprogramming to totipotency in embryos. J. Cell Biol. *218*, 70–82.

Lawson, K.A., Dunn, N.R., Roelen, B.A., Zeinstra, L.M., Davis, A.M., Wright, C.V., Korving, J.P., and Hogan, B.L. (1999). Bmp4 is required for the generation of primordial germ cells in the mouse embryo. Genes Dev. *13*, 424–436.

Lee, C.S., Lu, T., and Seydoux, G. (2017). Nanos promotes epigenetic reprograming of the germline by down-regulation of the THAP transcription factor LIN-15B. eLife *6*, e30201.

Levine, E., Cupp, A.S., Miyashiro, L., and Skinner, M.K. (2000). Role of transforming growth factor-alpha and the epidermal growth factor receptor in embryonic rat testis development. Biol. Reprod. *62*, 477–490.

Lu, F., Liu, Y., Inoue, A., Suzuki, T., Zhao, K., and Zhang, Y. (2016). Establishing chromatin regulatory landscape during mouse preimplantation development. Cell *165*, 1375–1388.

Macleod, D., Clark, V.H., and Bird, A. (1999). Absence of genome-wide changes in DNA methylation during development of the zebrafish. Nat. Genet. *23*, 139–140.

McKenna, A., Findlay, G.M., Gagnon, J.A., Horwitz, M.S., Schier, A.F., and Shendure, J. (2016). Whole-organism lineage tracing by combinatorial and cumulative genome editing. Science *353*, aaf7907.

Mello, C.C., Schubert, C., Draper, B., Zhang, W., Lobel, R., and Priess, J.R. (1996). The PIE-1 protein and germline specification in C. elegans embryos. Nature *382*, 710–712.

Merkhofer, E.C., Hu, P., and Johnson, T.L. (2014). Introduction to cotranscriptional RNA splicing. Methods Mol. Biol. *1126*, 83–96.

Middleton, R., Gao, D., Thomas, A., Singh, B., Au, A., Wong, J.J., Bomane, A., Cosson, B., Eyras, E., Rasko, J.E.J., and Ritchie, W. (2017). IRFinder: assessing the impact of intron retention on mammalian gene expression. Genome Biol. *18*, 51.

Mishima, Y., Giraldez, A.J., Takeda, Y., Fujiwara, T., Sakamoto, H., Schier, A.F., and Inoue, K. (2006). Differential regulation of germline mRNAs in soma and germ cells by zebrafish miR-430. Curr. Biol. *16*, 2135–2142.

Murphy, P.J., Wu, S.F., James, C.R., Wike, C.L., and Cairns, B.R. (2018). Placeholder nucleosomes underlie germline-to-embryo DNA methylation reprogramming. Cell *172*, 993–1006.e13.

Nakamura, A., Sato, K., and Hanyu-Nakamura, K. (2004). Drosophila cup is an eIF4E binding protein that associates with Bruno and regulates oskar mRNA translation in oogenesis. Dev. Cell *6*, 69–78.

Ohinata, Y., Ohta, H., Shigeta, M., Yamanaka, K., Wakayama, T., and Saitou, M. (2009). A signaling principle for the specification of the germ cell lineage in mice. Cell *137*, 571–584.

Ortega-Recalde, O., Day, R.C., Gemmell, N.J., and Hore, T.A. (2019). Zebrafish preserve global germline DNA methylation while sex-linked rDNA is amplified and demethylated during feminisation. Nat. Commun. *10*, 3053.

Oulhen, N., and Wessel, G.M. (2016). Differential Nanos 2 protein stability results in selective germ cell accumulation in the sea urchin. Dev. Biol. *418*, 146–156.

Ouyang, J.P.T., Folkmann, A., Bernard, L., Lee, C.Y., Seroussi, U., Charlesworth, A.G., Claycomb, J.M., and Seydoux, G. (2019). P granules protect RNA interference genes from silencing by piRNAs. Dev. Cell *50*, 716–728.e6.

Perino, M., and Veenstra, G.J. (2016). Chromatin control of developmental dynamics and plasticity. Dev. Cell *38*, 610–620.

Raj, B., Wagner, D.E., McKenna, A., Pandey, S., Klein, A.M., Shendure, J., Gagnon, J.A., and Schier, A.F. (2018). Simultaneous single-cell profiling of lineages and cell types in the vertebrate brain. Nat. Biotechnol. *36*, 442–450.

Raz, E. (2003). Primordial germ-cell development: the zebrafish perspective. Nat. Rev. Genet. *4*, 690–700.

Rechtsteiner, A., Ercan, S., Takasaki, T., Phippen, T.M., Egelhofer, T.A., Wang, W., Kimura, H., Lieb, J.D., and Strome, S. (2010). The histone H3K36 methyltransferase MES-4 acts epigenetically to transmit the memory of germline gene expression to progeny. PLoS Genet. *6*, e1001091.

Riemer, S., Bontems, F., Krishnakumar, P., Gömann, J., and Dosch, R. (2015). A functional Bucky ball-GFP transgene visualizes germ plasm in living zebrafish. Gene Expr. Patterns *18*, 44–52.

Roovers, E.F., Kaaij, L.J.T., Redl, S., Bronkhorst, A.W., Wiebrands, K., de Jesus Domingues, A.M., Huang, H.-Y., Han, C.-T., Riemer, S., Dosch, R., et al. (2018). Tdrd6a regulates the aggregation of Buc into functional subcellular compartments that drive germ cell specification. Dev. Cell *46*, 285–301.e9.

Rothschild, S.C., Lahvic, J., Francescatto, L., McLeod, J.J., Burgess, S.M., and Tombes, R.M. (2013). CaMK-II activation is essential for zebrafish inner ear development and acts through delta-Notch signaling. Dev. Biol. *381*, 179–188.

Saitou, M., and Yamaji, M. (2012). Primordial germ cells in mice. Cold Spring Harbor Perspect. Biol. *4*, a008375.

Schindelin, J., Arganda-Carreras, I., Frise, E., Kaynig, V., Longair, M., Pietzsch, T., Preibisch, S., Rueden, C., Saalfeld, S., Schmid, B., et al. (2012). Fiji: an open-source platform for biological-image analysis. Nat. Methods *9*, 676–682.

Seydoux, G., and Braun, R.E. (2006). Pathway to totipotency: lessons from germ cells. Cell *127*, 891–904.

Siddall, N.A., McLaughlin, E.A., Marriner, N.L., and Hime, G.R. (2006). The RNA-binding protein Musashi is required intrinsically to maintain stem cell identity. Proc. Natl. Acad. Sci. USA *103*, 8402–8407.

Sienski, G., Dönertas, D., and Brennecke, J. (2012). Transcriptional silencing of transposons by Piwi and maelstrom and its impact on chromatin state and gene expression. Cell *151*, 964–980.

Skvortsova, K., Tarbashevich, K., Stehling, M., Lister, R., Irimia, M., Raz, E., and Bogdanovic, O. (2019). Retention of paternal DNA methylome in the developing zebrafish germline. Nat. Commun. *10*, 3054.

Strasser, M.J., Mackenzie, N.C., Dumstrei, K., Nakkrasae, L.-I., Stebler, J., and Raz, E. (2008). Control over the morphology and segregation of zebrafish germ cell granules during embryonic development. BMC Dev. Biol. *8*, 58.

Strome, S., Kelly, W.G., Ercan, S., and Lieb, J.D. (2014). Regulation of the X chromosomes in Caenorhabditis elegans. Cold Spring Harbor Perspect. Biol. *6*, a018366.

Strome, S., and Updike, D. (2015). Specifying and protecting germ cell fate. Nat. Rev. Mol. Cell Biol. *16*, 406–416.

Suzuki, A., Saba, R., Miyoshi, K., Morita, Y., and Saga, Y. (2012). Interaction between NANOS2 and the CCR4-NOT deadenylation complex is essential for male germ cell development in mouse. PLoS One *7*, e33558.

Suzuki, H., Saba, R., Sada, A., and Saga, Y. (2010). The Nanos3-3'UTR is required for germ cell specific NANOS3 expression in mouse embryos. PLoS One *5*, e9300.

Tada, H., Mochii, M., Orii, H., and Watanabe, K. (2012). Ectopic formation of primordial germ cells by transplantation of the germ plasm: direct evidence for germ cell determinant in Xenopus. Dev. Biol. *371*, 86–93.

Tang, W.W., Dietmann, S., Irie, N., Leitch, H.G., Floros, V.I., Bradshaw, C.R., Hackett, J.A., Chinnery, P.F., and Surani, M.A. (2015). A unique gene regulatory network resets the human germline epigenome for development. Cell *161*, 1453–1467.

Updike, D.L., Hachey, S.J., Kreher, J., and Strome, S. (2011). P granules extend the nuclear pore complex environment in the C. elegans germ line. J. Cell Biol. *192*, 939–948.

Weidinger, G., Stebler, J., Slanchev, K., Dumstrei, K., Wise, C., Lovell-Badge, R., Thisse, C., Thisse, B., and Raz, E. (2003). dead end, a novel vertebrate germ plasm component, is required for zebrafish primordial germ cell migration and survival. Curr. Biol. *13*, 1429–1434.

Weidinger, G., Wolke, U., Köprunner, M., Klinger, M., and Raz, E. (1999). Identification of tissues and patterning events required for distinct steps in early migration of zebrafish primordial germ cells. Development *126*, 5295–5307.

White, R.J., Collins, J.E., Sealy, I.M., Wali, N., Dooley, C.M., Digby, Z., Stemple, D.L., Murphy, D.N., Billis, K., Hourlier, T., et al. (2017). A high-resolution mRNA expression time course of embryonic development in zebrafish. Elife *6*, e30860.

Wilhelm, J.E., Hilton, M., Amos, Q., and Henzel, W.J. (2003). Cup is an eIF4E binding protein required for both the translational repression of oskar and the recruitment of Barentsz. J. Cell Biol. *163*, 1197–1204.

Williamson, A., and Lehmann, R. (1996). Germ cell development in Drosophila. Annu. Rev. Cell Dev. Biol. *12*, 365–391.

Ying, Y., Qi, X., and Zhao, G.Q. (2001). Induction of primordial germ cells from murine epiblasts by synergistic action of BMP4 and BMP8B signaling pathways. Proc. Natl. Acad. Sci. USA *98*, 7858–7862.

Yoon, C., Kawakami, K., and Hopkins, N. (1997). Zebrafish vasa homologue RNA is localized to the cleavage planes of 2- and 4-cell-stage embryos and is expressed in the primordial germ cells. Development *124*, 3157–3165.

Zhang, W., Liu, Y., Zhang, M., Zhu, C., and Lu, Y. (2017). Quantitative reproducibility analysis for identifying reproducible targets from high-throughput experiments. BMC Syst. Biol. *11*, 73.

CellPress
OPEN ACCESS

## STAR★METHODS

### KEY RESOURCES TABLE

| REAGENT or RESOURCE | SOURCE | IDENTIFIER |
|---|---|---|
| Antibodies | | |
| Anti-Digoxigenin-POD Fab fragments | Roche, UK | Cat#11207733910; RRID: AB_514500 |
| Bacterial and Virus Strains | | |
| Alpha-selected competent cells | Bioline, UK | Cat#Bio-85026 |
| Chemicals, Peptides, and Recombinant Proteins | | |
| Triptolide | Sigma-Aldrich, UK | Cat#T3652 |
| Hepes | Sigma-Aldrich, UK | Cat#H3375 |
| Phenol Red | Sigma-Aldrich, UK | Cat#P0290 |
| Paraformaldehyde | Alpha Aesar, USA | Cat#43368 |
| Tricaine | Sigma-Aldrich, UK | Cat#E10521 |
| Critical Commercial Assays | | |
| RNeasy micro Kit | Qiagen, UK | Cat#74044 |
| Superscript IV | Thermo Scientific, UK | Cat#18090050 |
| mMESSAGE mMACHINE | Thermo Scientific, UK | Cat#AM1340 |
| TSA Plus Cyanine 3 System | Perkin Elmer, UK | Cat#NEL744001KT |
| DIG wash and block buffer set | Roche, UK | Cat#11585762001 |
| EZ DNA Methylation Gold kit | Zymo Research, USA | Cat#D5005 |
| GenomiPhi V2 DNA Amplification Kit | Cytiva, USA | Cat# 25660030 |
| SMART-Seq. v4 | Takara Bio Europe | Cat#634889 |
| Nextera XT library preparation kit | Illumina, UK | Cat#FC-131-1024 |
| Nextera DNA Sample Preparation Kit | Illumina, UK | Cat#FC-121-1030 |
| NextSeq 500/550 High Output 150 | Illumina, UK | Cat#20024906 |
| HiSeq SBS Kit v4 | Illumina, UK | Cat#FC-401-4002 |
| Deposited Data | | |
| RNA-seq | This study | E-MTAB-8707 |
| RNA-seq rescue | This study | E-MTAB-9857 |
| ATAC-seq | This study | E-MTAB-8741 |
| RRB-seq | This study | E-MTAB-9858 |
| Experimental Models: Organisms/Strains | | |
| Zebrafish: wildtype AB strain | N/A | N/A |
| Zebrafish: *tg(buc:egfp)* | Riemer et al., 2015 | ZDB-TGCONSTRCT-151209-1 |
| Zebrafish: *tg(kop:egfp)* | Blaser et al., 2005 | ZFIN: ZDB-TGCONSTRCT-161118-1 |
| Oligonucleotides | | |
| miR-430 Morpholino (target sequence) CACACGCATCTTGTTGTCTGCTGTT | Hadzhiev et al., 2019 | Gene Tools custom order |
| miR-430 Morpholino (mismatch) CCCACTCATATTGTTGTATGCTTTT | Hadzhiev et al., 2019 | Gene Tools custom order |
| Tdrd7 Morpholino (target sequence) AACCAACTCCACGTCACTCATCCTG | Strasser et al., 2008 | Gene Tools custom order |
| Tdrd7 Morpholino (mismatch) ACCCAACTGCACGCCACTAATACTG | Strasser et al., 2008 | Gene Tools custom order |
| PCR forward primer for zebrafish *dnd1* geneTTCACTCTTCATGGCTCGTG | This study | N/A |
| PCR reverse primer for zebrafish *dnd1* gene GTCAACAGACTCGGCTCTCC | This study | N/A |

(*Continued on next page*)

*Continued*

| REAGENT or RESOURCE | SOURCE | IDENTIFIER |
|---|---|---|
| PCR forward primer for zebrafish *nanos3* geneAGACTGAGGCCGT GTACACCTCTCACTACT | This study | N/A |
| PCR reverse primer for zebrafish *nanos3* geneGAGCAGTAGTTCTTGTCCACCATCG | This study | N/A |
| PCR forward primer for zebrafish *ddx4* gene AGGATCCTTCAAGAGCGATGA | This study | N/A |
| PCR reverse primer for zebrafish *ddx4* gene GGTATTGAAGAAGCTCGCACA | This study | N/A |
| PCR forward primer for zebrafish *myl12.1* geneCCAAGGTAAAGCTGCACTGT | This study | N/A |
| PCR reverse primer for zebrafish *myl12.1* geneCCACGAGAGCCCTGAACTTA | This study | N/A |
| PCR forward primer for zebrafish *tdrd7* geneTCTACCCAGCGGAAGCTTTA | This study | N/A |
| PCR reverse primer for zebrafish *tdrd7* gene CTGGTGTCCCACTGGTCTTT | This study | N/A |
| PCR forward primer for zebrafish *tdrd9* geneGGTCTCCGATCCGTAATCAG | This study | N/A |
| PCR reverse primer for zebrafish *tdrd9* gene AGCCTCCATCTCATCAAAGC | This study | N/A |
| Patch Bisulfite PCR primers | This study | Table S7 |
| Software and Algorithms | | |
| Fiji | Schindelin et al., 2012 | https://fiji.sc/ |
| Zen software | Zeiss | https://www.zeiss.com/microscopy/int/products/microscope-software/zen.html |

## RESOURCE AVAILABILITY

### Lead contact

Further information and requests for resources and should be directed to and will be fulfilled by the Lead Contact, Ferenc Mueller (F.Mueller@bham.ac.uk)

### Materials availability

This study did not generate new unique reagents or transgenic animals

### Data and code availability

The accession numbers for the datasets reported in this paper are ArrayExpress: E-MTAB-8707, ArrayExpress: E-MTAB-8741, ArrayExpress: E-MTAB-9857 and ArrayExpress: E-MTAB-9858.

Custom code used to analyse the reported data is available at https://github.com/fabiodorazio

## EXPERIMENTAL MODEL AND SUBJECT DETAILS

### Zebrafish

Wild type (AB) and Tg(Buc-GFP) lines were used for the experiments. The data shown during this work is obtained from embryos up to prim-5 stage (24 hours post fertilisation). All animal work was performed under the Project Licence # b6b8b391, in accordance with the UK Home Office regulations and UK Animals (Scientific Procedures) Act 1986. Up to eight pairs of adult zebrafish were kept in 3.5 litre polycarbonate tanks in a ZebTEC recirculating housing system (Techniplast, UK) with water temperature at 26°C. Adults were fed at least three times a day with a combination of brine shrimp cysts and ZM Medium Premium Granular dry food (ZMSystems, UK) and kept on a light-dark cycle of 14 and 8 hours.

Fish pairs were crossed in 1 litre breeding tanks and kept overnight separated. The next morning, the gate was removed and the eggs collected at intervals of 5 minutes to ensure synchrony. Fertilised eggs were dechorionated by 10mg/mL Pronase and serial washes in sterile E3 medium. After dechorionation, embryos were kept in agarose-coated petri dishes at 28.5°C in a 14/10 hours of light/dark respectively.

## METHOD DETAILS

### Microinjections of zebrafish embryos

Transient knock-down was achieved in zebrafish embryos through injection of mRNA-targeting Morpholinos. Phenotype rescue experiment were performed by injection of morpholino insensitive, *in-vitro* transcribed mRNA. Morpholinos from Strasser et al. (2008) were diluted in phenol red and about 0.3 pM were injected into the yolk of one-cell stage zebrafish embryos with a glass needle as described in Hadzhiev et al. (2019). Morpholino insensitive *tdrd7* mRNA for morpholino rescue was injected into the yolk of one-cell stage zebrafish embryos with a glass needle (600 ng/μl).

### Production of *tdrd7* RNA

Full-length *tdrd7* containing plasmid (A586.197Arescue-3'UTR197A) was linearized with NotI. RNA was produced under the sp6 promoter using the mMessage mMachine sp6 transcription kit (ThermoFisher scientific) following the manufacturerś instructions. RNA was purified with the Monarch RNA Cleanup Kit (NEB) and diluted for injections.

### Transcription block

Transcription block was achieved through embryo incubation in 1 μM triptolide (Sigma T3652) in E3 medium from the single-cell stage to completion of the experiment.

### PGC preparation for FACS

PGCs were isolated at different stages via FACS. Tg(Buc-GFP) heterozygous embryos were grown at the desired stage by incubation in E3 medium supplemented with 1mg/ml gentamicin at 28.5°C. Embryos were washed three times in sterile water before collection and about 200 of them were pulled in single microcentrifuge tubes. 500 μl of HBSS supplemented with 0.25% BSA and 10mM Hepes were added and dissociation occurred by pipetting for 2 minutes with a glass pipette. Excess of yolk was removed by two rounds of 3 minutes centrifugation at 350 x g at 4°C, while pelleted cells were resuspended in 1ml HBSS supplemented with 0.25% BSA and 10mM Hepes prior of filtering. Cell suspension was kept on ice for the entire isolation procedure.

### Fluorescent in-situ hybridization

Dechorionated embryos were collected at the desired stage, washed in cold PBS and fixed in 4% PFA at 4°C for 1 hour. Fixed embryos were then dehydrated in increasing dilutions of methanol (25, 50, 75, 100 %) and left from overnight to one month at -20°C. The embryos were rehydrated in decreasing dilutions of methanol (75, 50, 25%) and washed 5 times in PBST (0.1%) for 5 minutes with gentle agitation. In order to acclimatise the sample to the high temperature and the hybridization conditions, the embryos were incubated for 2 hours at 70°C in 200 μl of Hybridization Buffer (HB) (50% deionized formamide, 5X SSC, 0.1% Tween-20, 50 μg/ml of heparin bile salts, 500 μg/ml of extracted RNase-free tRNA, pH 6.0). From 50 to 100 ng of DIG-labelled RNA probes targeting *dazl* transcripts (Forward: ACTAAAGTTGTAGCTGGGCCT, Reverse: CCTGAGTGGGCGTTAATGTT) were added to the HB and incubated overnight at 70°C. The next day, the probes were removed by four washes in increasing dilutions of 2 X SSC (NaCl 0.3M; Sodium citrate 0.03M) at 70°C (25, 50, 75, 100 %). The sample was then washed twice in 0.2% of SSC at 70°C 30 minutes each. The 0.2 X SSC was replaced by four serial dilutions in PBST 0.1% in order to remove any left-over probe. Washes were performed at room temperature with gentle agitation. The embryos were blocked in Blocking Buffer + Maleic Acid (Roche, 11585762001) for at least 3 hours before the anti-DIG antibody was added in a concentration of 1:5000 and incubated overnight at 4°C. The next day the antibody was washed five times in PBST 0.1% with gentle agitation at room temperature (30 minutes each) and fluorescently-tagged by horseradish peroxidase-catalysed signal amplification (Thermo Scientific, q

### Imaging

Embryos were placed in an agarose-coated petri dish and eventually embedded in agarose and imaged with a Zeiss 780 confocal or Z1 light sheet microscopes. Images were taken with the Zeiss ZEN pro 2.0 acquisition software with standard settings. Fixed samples were mounted in glycerol-based VectaShield (Vector laboratories, H-1000, UK) on a slide and covered with a glass slip. When preservation of the body shape was required, imaging dishes with glass bottom were used to avoid disintegration of the embryos.

### Nuclear-cytoplasmic fractionation and qPCR

Embryos from three biological repeats were set on ice and dissociated as described earlier. Nuclear and cytoplasmic fractions were separated by two washes in nuclei isolation buffer (Tris-HCl pH 7.4 10mM, NaCl 10mM, MgCl2 3mM and 0.1% IGEPAL CA-630) followed by 5 minutes centrifugation at 500 x g. RNA was extracted from the two fractions with the RNAeasy Mini Extraction kit (Qiagen, 74044, UK), converted in cDNA via the SuperScript™ III Reverse Transcriptase (Thermo Fisher, 18080093, UK) and used for qPCR.

### Reduced representation bisulfite sequencing (RRBS-Seq)

Genomic DNA were extracted from 4500-7500 sorted somatic cells or PGCs at high, dome and prim-5 stage in duplicates and digested with MspI at 37°C for 3 hours. The fragment ends were repaired with Klenow exo at 37°C for 50 minutes. Then Methylated Illumina Pair-end Adaptors were ligated to gDNA fragments using T4 DNA ligase. The bisulfite conversion were performed using

Zymo Research EZ DNA Methylation Gold kit. Libraries were PCR amplified for twenty cycles using Platinum Taq DNA polymerase and sequenced on Illumina HiSeq 2500 on 50bp single-end mode.

### Patch bisulfite PCR

Genomic DNA were extracted from 5000 sorted somatic cells or PGCs at prim-5, with three replicates for each cell types. Whole Genome Amplified genomic DNA (WGA gDNA) were generated from prim-5 WT Tu fish embryos using GE GenomiPhi V2 DNA Amplification Kits. 30 ng of DNA from sorted cells, 100 ng of genomic DNA from prim-5 embryos (non-bisulfite conversion control) and 100 ng of WGA gDNA (hypomethylation control) were digested with HpyCH4V and NlaIII at 37°C for 1 hour following by heat inactivation for 20 minutes at 65°C. Then custom universal primers were ligated to the targeted fragments using HiFi Taq DNA ligase with the help of gene specific designed oligo patches. The reaction was incubated at 95°C for 15 minutes followed by 30 seconds at 94°C and 4 minutes at 65°C for 25 cycles, and was held at 4°C. Unligated DNA fragments were removed by Exo I and Exo III treatment at 37°C for 1 hour followed by heat inactivation at 80°C for 20 minutes. Bisulfite Conversion were performed following Zymo EZ DNA Methylation Gold kit manufacturer's instruction. This step was skipped for non-bisulfite control sample. The eluted DNA were PCR amplified using EpiMark Hot Start Taq DNA polymerase. Libraries were sequenced on Miseq 250bp paired-end mode.

### ATAC-seq library preparation and sequencing

Two biological repeats for PGCs and somatic cells at high (wild type), prim-5 (wild type) and morpholino-injected prim-5 (5mm and MO) were prepared. Cells were sorted into 500 μl of cold PBS Mg-, Ca- and immediately treated for ATAC. Nuclear isolation and transposition reaction occurred as described in Buenrostro et al. (2013). In brief, cells were collected in Hank's Buffer after FACS. Nuclei were extracted by pipetting cells up and down in lysis buffer (10 mM Tris-HCl, pH 7.4, 10 mM NaCl, 3 mM MgCl2, 0.1% IGEPAL CA-630) and pelleted in a cold centrifuge for 10 minutes at 500 x g. TD Buffer and Tn5 Transposase from the Nextera$^{TM}$ DNA Sample Prep Kit (Illumina, FC-121-1030, UK) were added to the nuclei and incubated at 37°C for 30 minutes. Tagmented DNA was purified by QIAGEN MinElute Clean up Kit, PCR amplified and libraries were purified with 1.2X volume of AMPure XP beads. DNA bound to the beads was washed twice in 80% ethanol and eluted in 20 μl of water. Indexed fragments were checked in concentration by qPCR, profiled by Bioanalyzer, equimolarly pooled and sequenced on an Illumina Next-Seq 550.

### RNA-seq library preparation and sequencing

cDNA was prepared from two biological repeats according to manufacturer's instructions as follows. Two hundred cells were sorted in 8.5μl of water (0.2U/μl RNase inhibitor). Immediately after collection, cells were added with 1μl of lysis buffer (0.2U/μl RNase inhibitor) and 1 ul of ERCC Mix1-2 (final dilution 1x10-6) and flash-frozen. Reverse transcription was performed following the SMART-Seq v4 protocol. In brief, frozen cells were thawed on ice for five minutes and 2 μl of 3' SMART-Seq CDS Primer II A were added to each sample. The reaction was pre-heated at 72°C for 3 minutes and the reverse transcription mix was added. cDNA was then amplified by 16-18 PCR cycles.

Indexed fragments were checked in concentration by qPCR, profiled by Bioanalyzer, equimolarly pooled and sequenced on an Illumina Next-Seq 550.

## QUANTIFICATION AND STATISTICAL ANALYSIS

### Image processing and analysis

Confocal and light sheet images were analysed using ZEN (Version pro 2.0) and Fiji (Version 2.0.0-rc-69/1.52p 2018) software. All images are shown as maximum intensity projections of multi-stacks acquisition. Brightness and contrast adjustments were applied to reduce signal to noise ratio.

Phenotypic analysis of Tdrd7 KD PGCs was performed as follows. Germ granules areas were automatically detected and measured by the Analyze tool in Fiji after median filter was applied. Germ granule-nucleus contact ratio was calculated by dividing the length of the perimeter segment adjacent to the nucleus for each granule by the total area of germ granules in a single cell. Regions of interest were manually drawn with the freehand tool in Fiji. Perimeter, area and signal intensity were measured with the Analyze tool.

### ATAC-seq analysis

Paired-end ATAC reads were mapped to the genome using Bowtie2, not allowing discordant mapping of reads (–no-discordant) and insert sizes larger than 5000bp (–maxins 5000). Results were filtered for mapping quality (10) and for mapping to chromosomes 1 to 25 with the exclusion of the mitochondrial chromosome and of contigs present in danRer7/10 assemblies. There was no removal of subsequent read pairs mapping to the same locus (so called duplicate removal).

Mapped and filtered ATAC reads were corrected for Tn5 transposase overhang by adding 5bp to the position of the start of the first read and by subtracting 4bp from the end of the second read in the read pair as described in Buenrostro et al. (2013). Both thus obtained Tn5 cut sites were extended by a fixed amount. For genome browser visualisation 25bp was added to Tn5 cut sites yielding two 51bp-long regions for each read pair. For PCA and SOM analysis a shorter 5bp extension was used. No selection for particular insert size fraction was used.

### Enhancer calling in somatic cells and PGCs

The set of putative enhancers was obtained from ATAC-seq in the following way. MACS2 was used with options -f BED -g 1.412e9 –keep-dup all –nolambda –nomodel to call peaks in each sample and replicate separately. Four replicate pairs in PGCs and somatic cells at high and at prim5 stages were used to identify peaks reproducible across replicates with Irreproducible Discovery Rate (IDR)2 approach. At 5% IDR the number of reproducible peaks were for soma/high: 12,862; PGC/high: 14,029; soma/prim5: 79,494; PGC/prim5: 61,641. After a further removal of peaks within 500bp from any known transcript start (ENSEMBL version 79/91) and of width greater than 1000bp remaining peak numbers dropped to soma/high: 4,935; PGC/high: 5,097; soma/prim5: 55,784; PGC/prim5: 36,071. Finally, four peaks sets were merged in a union and thus unified peaks were once again filtered for length <= 1000bp. This yielded a total of 70,612 candidate enhancers.

### Principal component analysis and self organising map clustering

In order to assign open chromatin scores to enhancers, windows of size 601bp around enhancer centre were used. ATAC signal levels from genome browser track were extracted (sum of signal values in 601bp bins proportional to the number of 5' ends of reads falling into these bins and normalised to the total number of reads in each sample) with the help of genomation package3 and saved into a matrix. For PCA and SOM, levels were log-transformed and "centred" by subtracting the mean of each matrix column (sample). For SOM, an additional row (enhancer) centering was performed effectively making SOM operate on log-fold change values.

### Bioinformatic analysis for RRBS

Fastq files were aligned to ZV10 genome and processed using bismark(–bowtie1). Methylation level data were collected using bismark_methylation_extractor with parameters of –bedGraph –cutoff 6 –merge_non_CpG –comprehensive. Following methylation data were analysed using methylKit package in R with three replicates for each cell types at different developmental stages.

### Bioinformatic analysis for Patch bisulfite PCR

Adapters were removed using cutadapt with parameters of -a AGTGTGGGAGGGTAGTTGGTGTT -A ACTCCCCACCTTCCTCATT CTCTAAGACGGTGT –minimum-length 10 for Read 1 and Read 2. Adapter trimmed fastq files were then aligned to ZV10 genome and processed using bismark (–bowtie2). Methylation level data were collected using bismark_methylation_extractor with parameters of –bedGraph –cutoff 6 –merge_non_CpG –comprehensive. The output CpG coverage files were converted to colorBED files using a custom script. The colorBED files were loaded and visualized on UCSC genome browser.

### RNA-seq analysis

Fastq files were checked for quality by fastqc and trimmed by trimmomatic. Sequencing reads were aligned to the zebrafish genome (danRer9/10) or to the ERCC reference file by STAR (v.2.6.1) with the following settings:

–quantMode GeneCounts –outFilterMultimapNmax 1000 –outSAMmultNmax 1000 –outFilterMismatchNmax 999 –outFilterMismatchNoverReadLmax 0.06 –alignSJoverhangMin 8 –alignSJDBoverhangMin 1 –outFilterType BySJout –alignIntronMin 20 –alignIntronMax 500000 –alignMatesGapMax 500000

Raw read counts were loaded into R and differential expression analysis over samples and stages was performed using DESeq2 (v.1.6.3) and maSigPro packages.

Zygotic genes were defined as those with expression lower than 2 tpm at 256-cell stage (before zygotic transcription starts), which show an increase in expression levels at the subsequent analysed developmental stage.

For measurement of absolute RNA amount, ERCC reads were normalised to rpkm and those smaller than 1 discarded. A linear regression curve was obtained for the expected concentrations of ERCC fragments and the obtained rpkm in each sample. The concentrations of each RNA were estimated through the linear regression equation: $y = a + bX$.

Intron retention analysis was performed by IRFinder (IRFinder-1.2.3) with standard settings. The IR ratio represents the intron sequencing depth divided by the maximum number of reads mapping to the 3' and 5' splice sites summed to the intron sequencing depth.

### Statistics

All experiments for which statistical analyses were performed were repeated three times. All sequencing experiments were performed in biological duplicates with the exception of ATAC-seq for dome and 10-somites stages. Data from independent biological repeats were pooled together and the statistical distribution of the dataset was evaluated by Shapiro-Wilk test. For normally distributed datasets, the p-value was estimated upon t-test, while Wilcoxon test was used for non-normally distributed datasets. Statistical methods used, p-values and sample population are indicated in figures, figure legends and in the Results. Data presented as boxplot report sample median and percentiles. Outliers are omitted for visual purposes but not excluded from statistical analyses. Bar charts report mean and error bars indicate standard error.

Developmental Cell, Volume *56*

## Supplemental information

# Germ cell differentiation requires Tdrd7-dependent chromatin and transcriptome reprogramming marked by germ plasm relocalization

**Fabio M. D'Orazio, Piotr J. Balwierz, Ada Jimenez González, Yixuan Guo, Benjamín Hernández-Rodríguez, Lucy Wheatley, Aleksandra Jasiulewicz, Yavor Hadzhiev, Juan M. Vaquerizas, Bradley Cairns, Boris Lenhard, and Ferenc Müller**

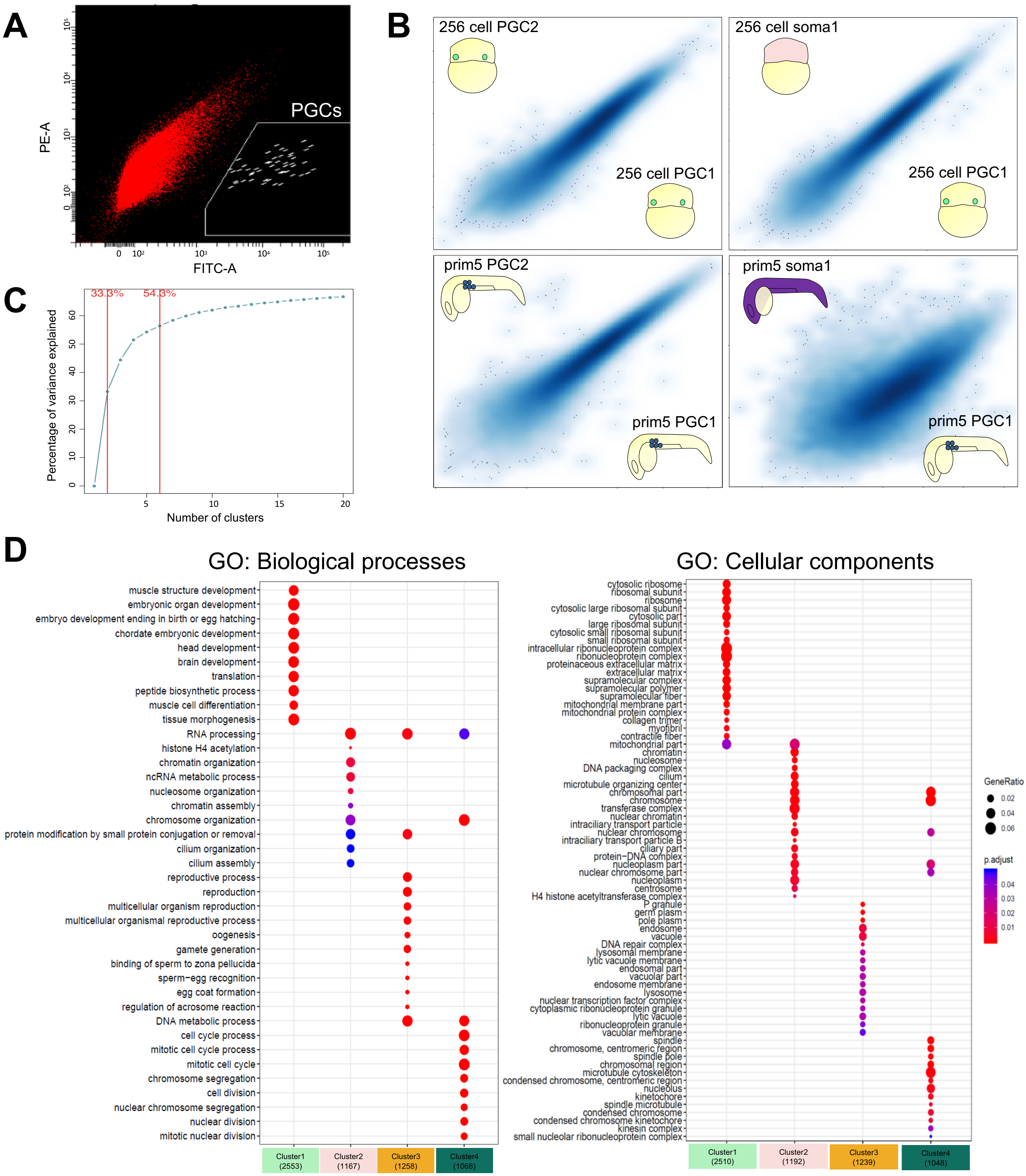


**Supplementary Figure 1**. **Characterisation of PGC transcriptome highlights early developmental similarities and late divergence between PGCs and somatic cells**. **Related to Figure 1** (A) FACS profile of GFP-expressing PGCs at prim-5 stage. GFP-positive and –negative cells are shown in white and red respectively. (B) Transcriptome correlation profiles after RNA-seq reads normalisation. Read counts for each gene were normalised for library size and gene length (tpm) and correlation among stages and cell types was compared. Reads are shown as $Log_2$(tpm+1). (C) Justification of number of k-means selected based on inter-sample variance. (D) Biological processes and cellular components GO analysis for the four identified expression groups by k-mean clustering (p-adjusted < 0.05).

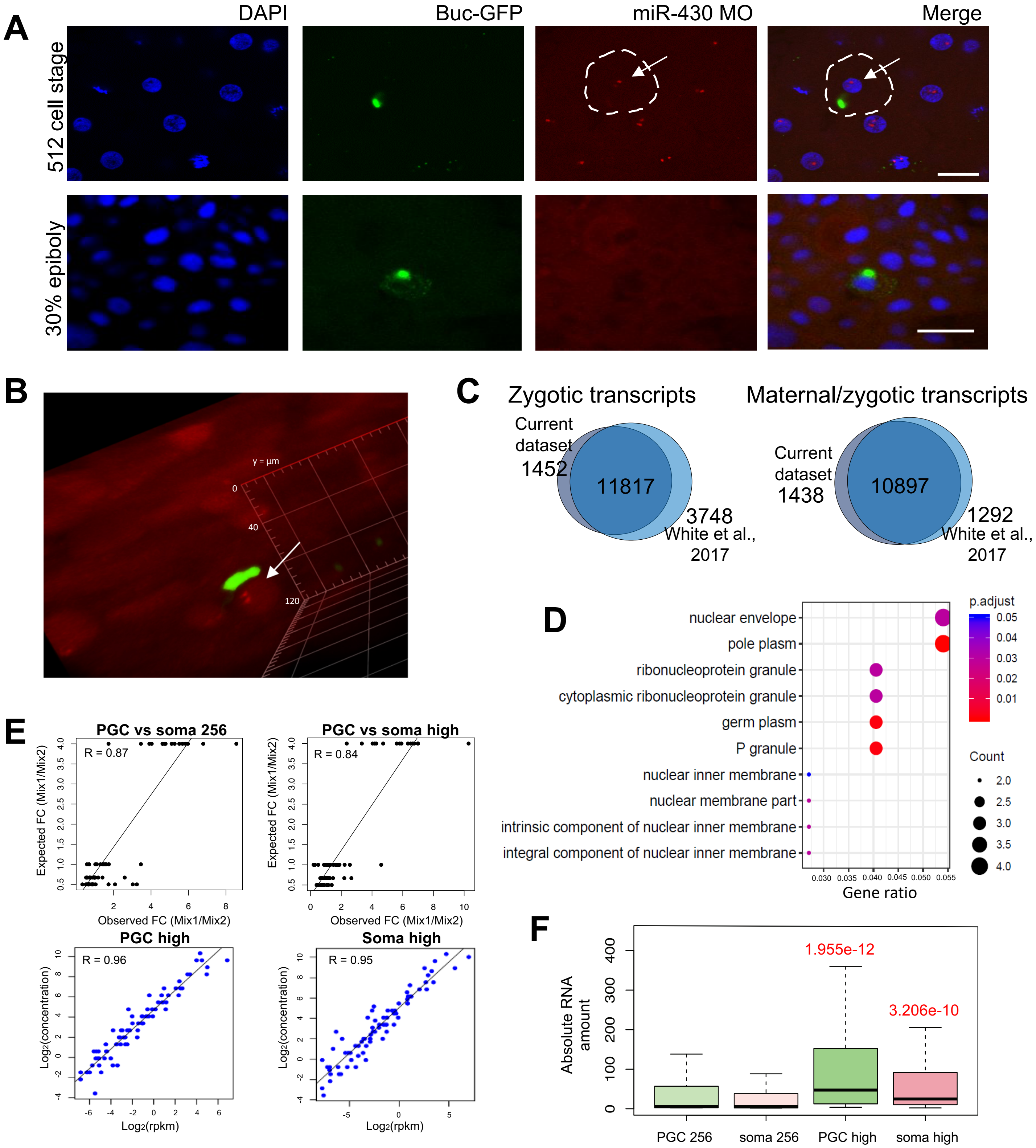


**Supplementary Figure 2. PGCs do not delay transcriptional activation. Related to Figure 2** (A) Maximum intensity projection of a multi-stack image of *miR-430* transcriptional activity at 512-cell stage and 30% epiboly. PGC boundaries are highlighted as white, dashed lines. Arrow indicates the nucleus of a germ plasm-carrying cell where transcriptional foci are detected. Scale bar is 30 µm. Number of embryos, n = 19 and 31 respectively. (B) 3D rendering of a *miR-430*-expressing nucleus (arrow) and a GFP-tagged germ granule. White arrow indicates the germ cell nucleus. Number of embryos, n = 24. (C) Venn diagram for predicted zygotic/maternal and zygotic genes from two independent RNA-seq datasets. (D) Cellular components GO analysis for genes upregulated in PGCs after the first wave of ZGA (from 256-cell to high stage). P-adjusted < 0.05. (E) Representative regression scatter plots between expected vs observed ERCC mix ratios (top) and ERCC concentrations vs ERCC reads (bottom). Tpm threshold for normalised ERCC reads is 1. (F) Absolute RNA concentration normalised to internal control (ERCC spike-in) for genes upregulated in germ plasm-carrying cells between 256-cell and high stage. Numbers show p-values calculated by Wilcoxon test.

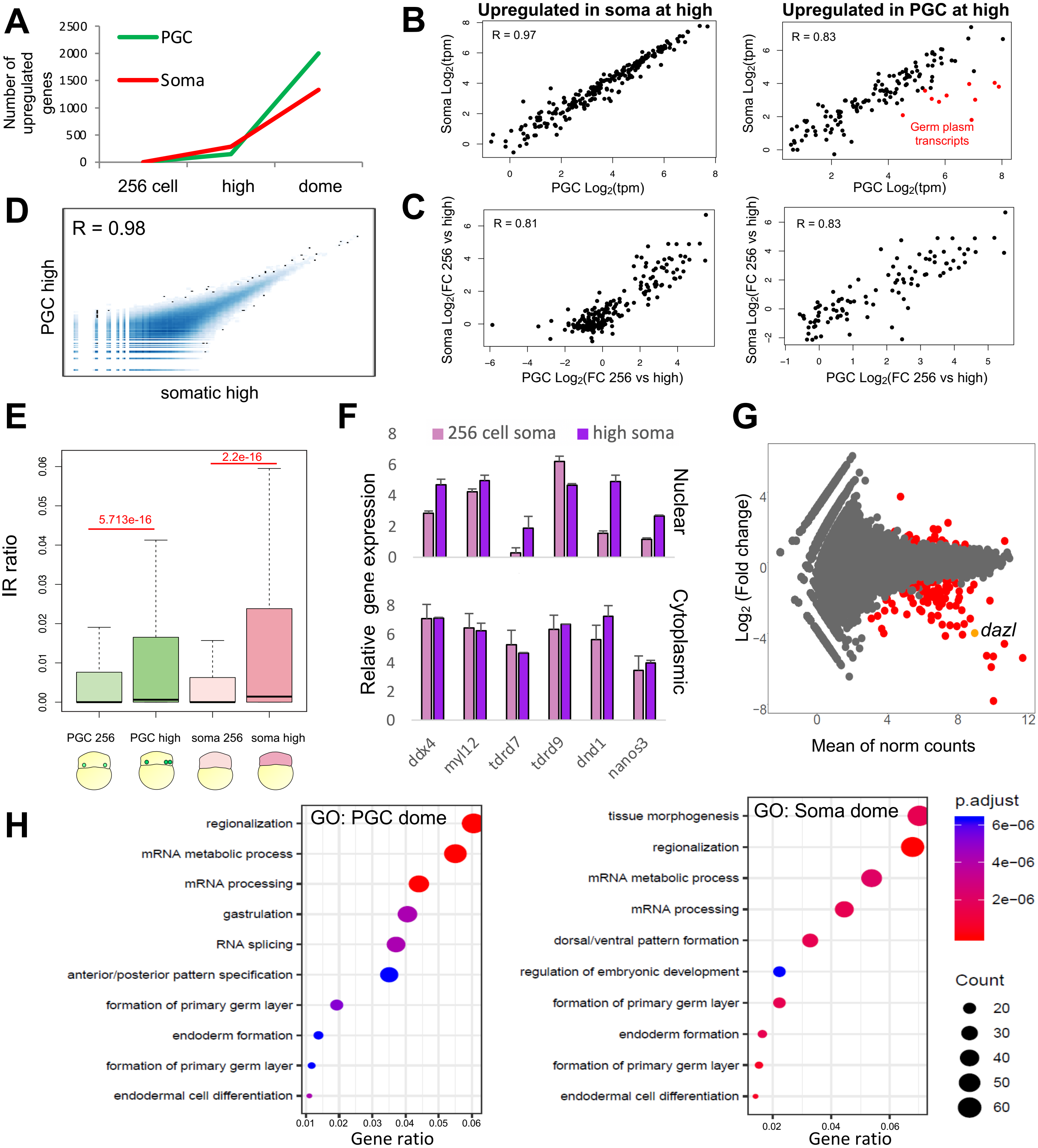


**Supplementary Figure 3. Differential transcriptome between PGCs and somatic cells at early stages is not caused by differential transcription. Related to Figure 3** (A) Count of upregulated genes from previous stage in PGCs and somatic cells at high and dome stages. (B) Correlation of normalised RNA-seq reads (tpm) for genes upregulated from the previous stage in the PGCs or somatic cells only at high stage. Germ plasm genes are in red. (C) Correlation between fold change increases from 256-cell to high stages in PGCs and somatic cells for gene subsets. (D) Correlation plot for open chromatin regions detected in PGCs and somatic cells at high stage. (E) Intron retention ratio for all transcripts in PGCs (green) and somatic cells (purple) before and after ZGA. T-test was used to calculate p-values (in red). (F) Relative gene expression fold change normalised to a reference gene in somatic cells for nuclear and cytoplasmic fractions (number of replicates = 2). Error bars show standard errors. (G) Differential expressed genes between 256-cell and high stages in PGCs. Significantly differentially expressed genes are in red. *dazl* is in orange. P adjusted < 0.1, $\log_2 FC < -1/> 1$. (H) Biological processes GO analysis for genes upregulated from high to dome.

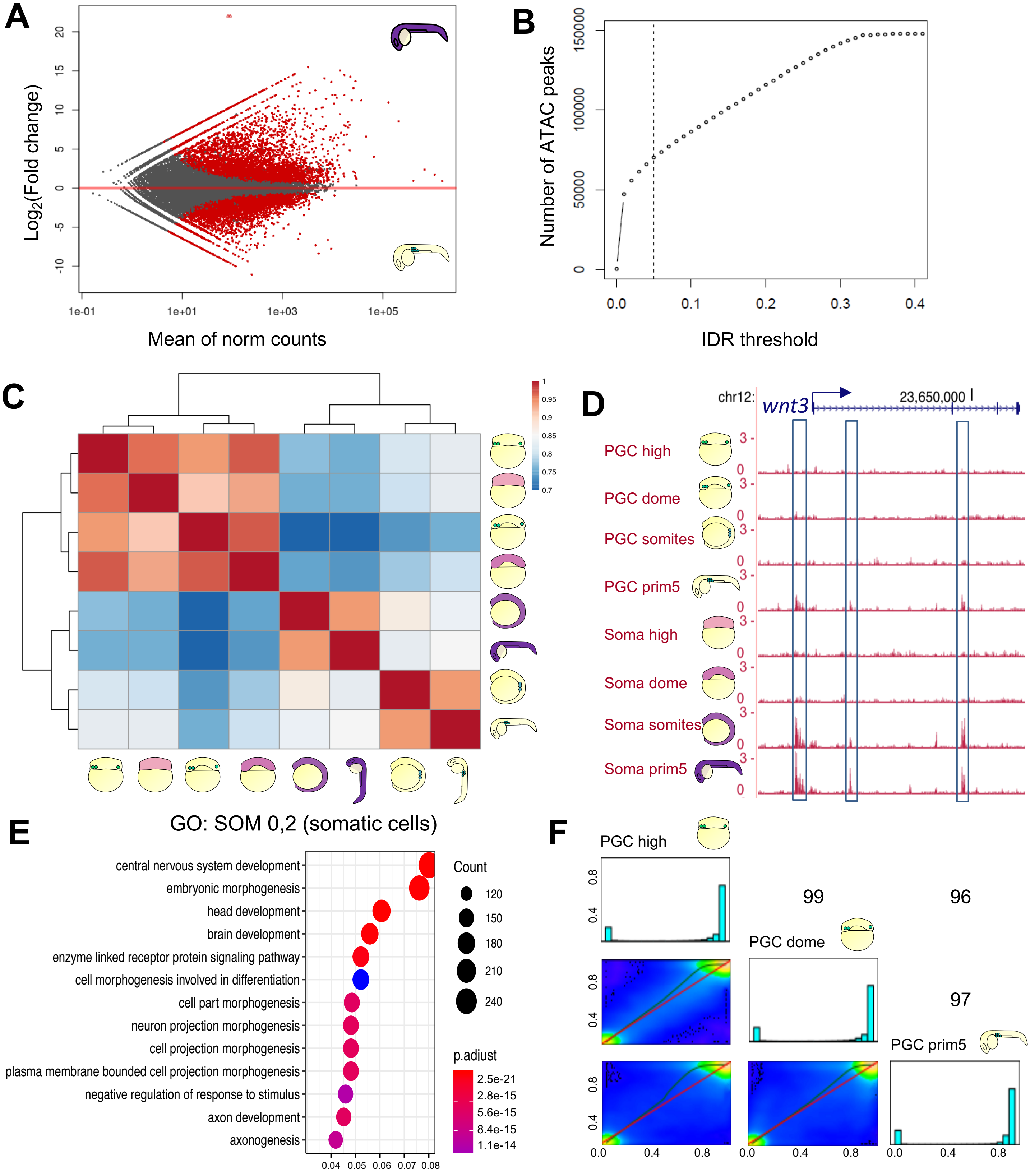


**Supplementary Figure 4. Gradual acquisition of germ identity is accompanied by epigenetic changes. Related to Figure 4** (A) Differentially expressed genes in PGCs and somatic cells at prim-5 stage. Significantly differentially expressed genes are in red. P adjusted < 0.1. (B) Reproducible ATAC peaks identified in function of the Irreproducible Discovery Rate (IDR) threshold. (C) Unsupervised Pearson correlation heatmap for developmental stages and cell types after selection of reproducible ATAC peaks. (D) Genome browser view of ATAC-seq tracks (magenta) of *wnt3* gene in PGCs and somatic cells across early development. Blue boxes highlight putative regulatory elements. Arrows show transcriptional directionality. (E) GO terms for genes associated with open chromatin regions upregulated in late somatic cells. (F) CpG base Pearson correlation shown as heatmap and histogram of CpG frequency.

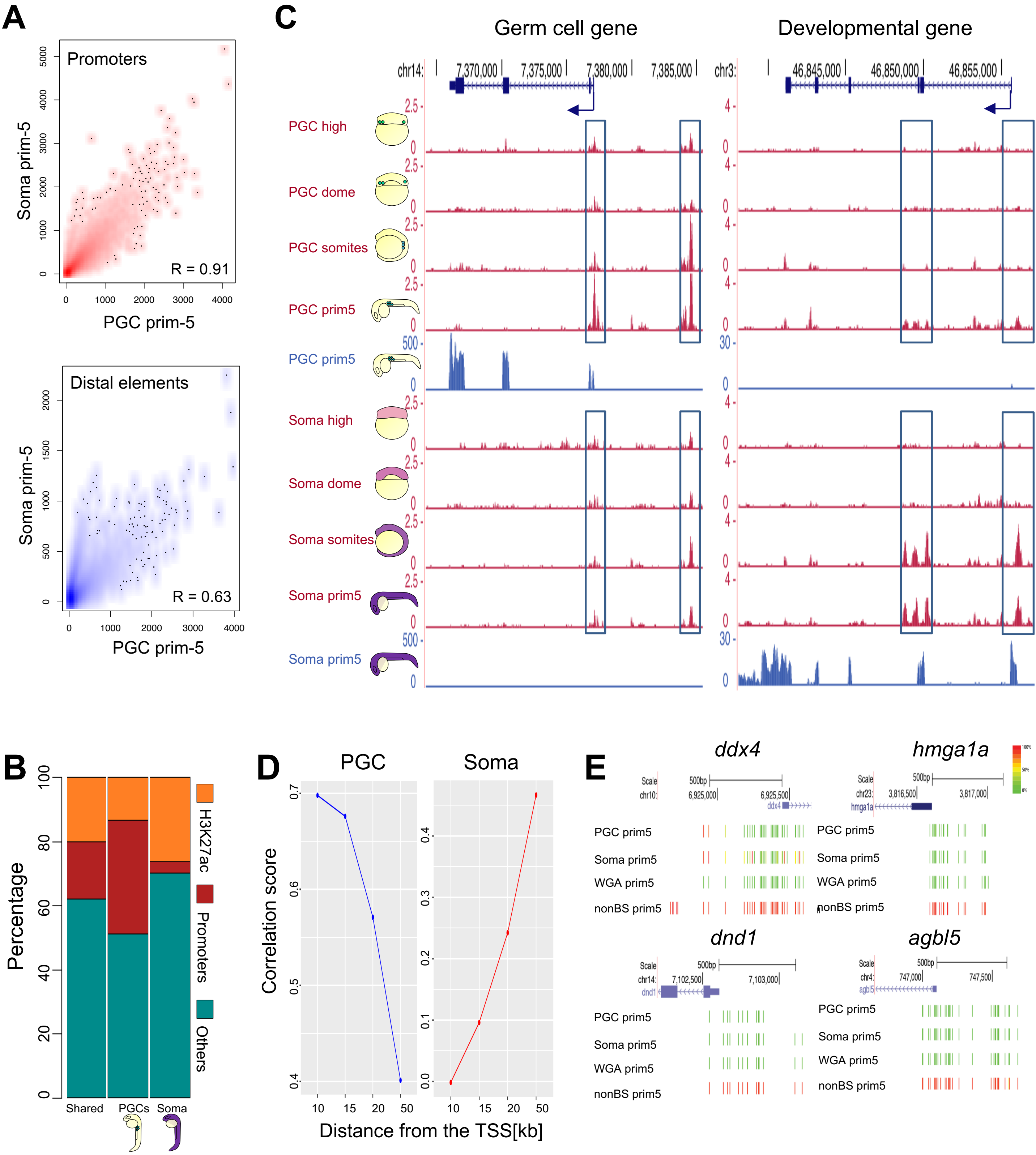


**Supplementary Figure 5. PGCs do not open chromatin at regions identified as putative enhancers. Related to Figure 5** (A) Correlation scatter plots of ATAC peaks between PGCs and somatic cells for promoters and distal elements at prim-5 stage. (B) Percentage of ATAC peaks associated with H3K27ac and promoters in PGCs and somatic cells at prim-5 stage. (C) Genome browser view of open chromatin regions (ATAC-seq in magenta) and transcript levels (RNA-seq in blue) in PGCs and somatic cells. Blue boxes highlight putative regulatory elements and promoters. Arrows show transcriptional directionality. (D) Correlation score between chromatin accessibility and gene transcription in function of distance from the TSS. Correlation score is represented as the absolute ATAC $log_2FC$ value associated to genes upregulated in PGCs (blue) or somatic cells (red) (E) Genome browser view of four representative promoters upregulated in late PGCs versus somatic cells. CpG methylation levels are reported as coloured bars.

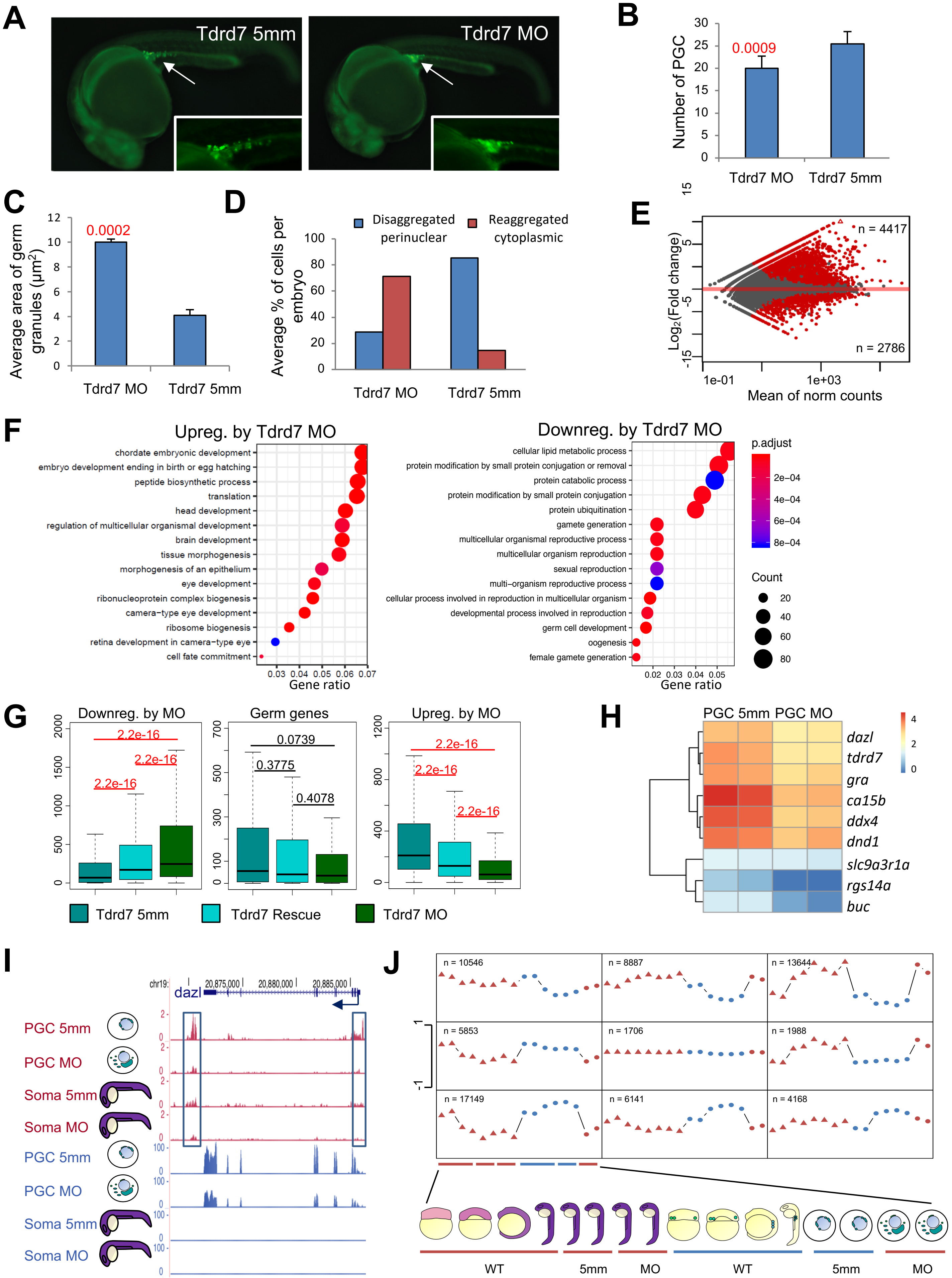

A
Tdrd7 5mm
Tdrd7 MO
B
Number of PGC
0.0009
Tdrd7 MO
Tdrd7 5mm
C
Average area of germ granules (μm²)
0.0002
Tdrd7 MO
Tdrd7 5mm
D
Disaggregated perinuclear
Reaggregated cytoplasmic
Average % of cells per embryo
Tdrd7 MO
Tdrd7 5mm
E
n = 4417
n = 2786
Log2(Fold change)
Mean of norm counts
F
Upreg. by Tdrd7 MO
chordate embryonic development
embryo development ending in birth or egg hatching
peptide biosynthetic process
translation
head development
regulation of multicellular organismal development
brain development
tissue morphogenesis
morphogenesis of an epithelium
eye development
ribonucleoprotein complex biogenesis
camera−type eye development
ribosome biogenesis
retina development in camera−type eye
cell fate commitment
Gene ratio
Downreg. by Tdrd7 MO
cellular lipid metabolic process
protein modification by small protein conjugation or removal
protein catabolic process
protein modification by small protein conjugation
protein ubiquitination
gamete generation
multicellular organismal reproductive process
multicellular organism reproduction
sexual reproduction
multi−organism reproductive process
cellular process involved in reproduction in multicellular organism
developmental process involved in reproduction
germ cell development
oogenesis
female gamete generation
Gene ratio
p.adjust
Count
G
Downreg. by MO
2.2e-16
Germ genes
0.0739
0.3775
0.4078
Upreg. by MO
Tdrd7 5mm
Tdrd7 Rescue
Tdrd7 MO
H
PGC 5mm
PGC MO
dazl
tdrd7
gra
ca15b
ddx4
dnd1
slc9a3r1a
rgs14a
buc
I
chr19:
dazl
20,875,000
20,880,000
20,885,000
PGC 5mm
PGC MO
Soma 5mm
Soma MO
PGC 5mm
PGC MO
Soma 5mm
Soma MO
J
n = 10546
n = 8887
n = 13644
n = 5853
n = 1706
n = 1988
n = 17149
n = 6141
n = 4168
WT
5mm
MO
WT
5mm
MO

**Supplementary Figure 6**. **Tdrd7 knock-down impacts germ plasm morphology and transcriptome. Related to Figure 6** (A) Fluorescent microscopy of Buc-GFP-expressing embryos at prim-5 stage injected with *tdrd7* 5mm and *tdrd7* MO oligos ($8 < n < 14$). (B) Number of Buc-GFP-expressing cells per embryo at prim-5 stage. P-value against control is shown ($10 < n < 15$, $p < 0.05$). Red colour indicates significant difference based on Wilcoxon test. (C) Measurement of germ granule size. P-value against control is shown in red ($9 < n < 12$, $p < 0.05$). Based on Wilcoxon test. (D) Assessment of germ plasm phenotype shown as percentage of cells per embryo ($10 < n < 15$). (E) Differential expression analysis for genes upregulated and downregulated by Tdrd7 KD in PGCs at prim-5 stage. Significantly differentially expressed genes are in red. P adjusted < 0.1, $\log_2$FC < -1/> 1. (F) GO terms for genes upregulated and downregulated by Tdrd7 translational inhibition in PGCs. (G) Box plots of normalised RNA-seq counts (tpm) for selected gene classes. Numbers show p-values in red colour if < 0.005 or black if > 0.005 based on Wilcoxon test. (H) Gene expression heatmap for germ plasm-localised transcripts. Colour intensities indicate $\log_2$(tpm+1). (I) Genome browser view of ATAC profiles after morpholino injections. Open chromatin (ATAC-seq) is shown in magenta and normalised transcript levels (RNA-seq) are shown in blue. Arrows show transcriptional directionality. (J) Self-organising map of reproducible open chromatin regions. Wild type (WT) PGCs and somatic cells are represented as circles and triangles respectively. Tdrd7 KD PGCs are shown as red circles.
